# Electrochemical Oxidation for Treatment of PFAS in Contaminated Water and Fractionated Foam—A Pilot-Scale Study

**DOI:** 10.1021/acsestwater.2c00660

**Published:** 2023-03-15

**Authors:** Sanne J. Smith, Melanie Lauria, Lutz Ahrens, Philip McCleaf, Patrik Hollman, Sofia Bjälkefur Seroka, Timo Hamers, Hans Peter H. Arp, Karin Wiberg

**Affiliations:** † Department of Aquatic Sciences and Assessment, Swedish University of Agricultural Sciences (SLU), P.O. Box 7050, SE-750 07 Uppsala, Sweden; ‡ Department of Environmental Science, Stockholm University, Svante Arrhenius Väg 8, 10691 Stockholm, Sweden; § Uppsala Water and Waste AB, P.O. Box 1444, SE-751 44 Uppsala, Sweden; ∥ Nova Diamant AB, Tryffelvägen 17, 75646 Uppsala, Sweden; ⊥ Amsterdam Institute for Life and Environment (A-LIFE), Vrije Universiteit Amsterdam, De Boelelaan 1085, 1081 HV Amsterdam, The Netherlands; # Norwegian Geotechnical Institute (NGI), P.O. Box 3930, Ullevål Stadion, NO-0806 Oslo, Norway; ¶ Department of Chemistry, Norwegian University of Science and Technology (NTNU), NO-7491 Trondheim, Norway

**Keywords:** foam fractionation, electrochemical oxidation, per- and polyfluoroalkyl substances, landfill leachate, groundwater, numerical modeling

## Abstract

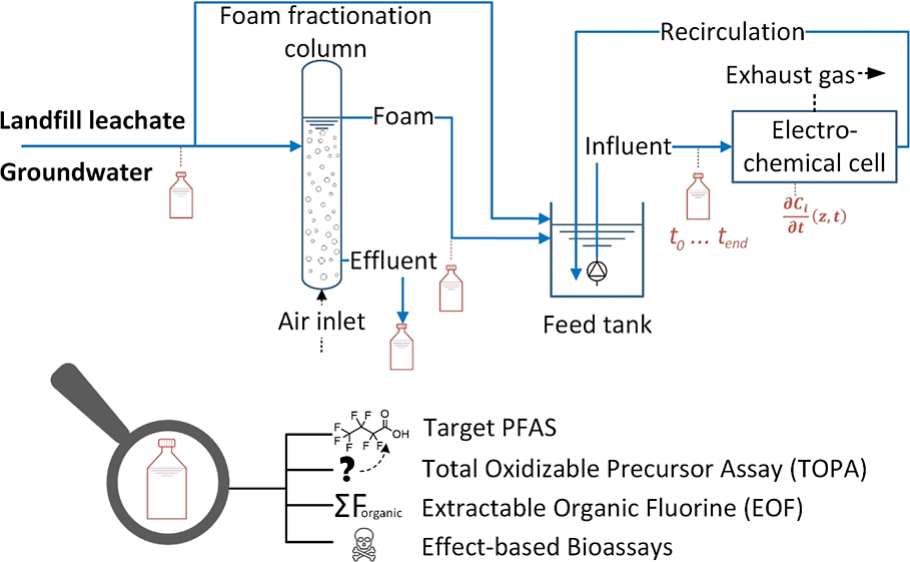

Per- and polyfluoroalkyl substances (PFAS) are persistent synthetic contaminants that are present globally in water and are exceptionally difficult to remove during conventional water treatment processes. Here, we demonstrate a practical treatment train that combines foam fractionation to concentrate PFAS from groundwater and landfill leachate, followed by an electrochemical oxidation (EO) step to degrade the PFAS. The study combined an up-scaled experimental approach with thorough characterization strategies, including target analysis, PFAS sum parameters, and toxicity testing. Additionally, the EO kinetics were successfully reproduced by a newly developed coupled numerical model. The mean total PFAS degradation over the designed treatment train reached 50%, with long- and short-chain PFAS degrading up to 86 and 31%, respectively. The treatment resulted in a decrease in the toxic potency of the water, as assessed by transthyretin binding and bacterial bioluminescence bioassays. Moreover, the extractable organofluorine concentration of the water decreased by up to 44%. Together, these findings provide an improved understanding of a promising and practical approach for on-site remediation of PFAS-contaminated water.

## Abstract 2

Foam fractionation followed by electrochemical oxidation is a promising treatment approach for PFAS-contaminated water. This study combined an up-scaled experimental approach with thorough analysis strategies to generate an improved understanding of this treatment train.

## Introduction

The widespread presence of per- and polyfluoroalkyl substances (PFAS), particularly in the aquatic environment, has become a global cause for concern.^1−4^ PFAS originate from various sources like the use of aqueous film-forming foam (AFFF), industrial releases, landfilling of PFAS-containing waste, and atmospheric deposition.^5−7^ Additionally, the breakdown of less mobile perfluoroalkyl acid (PFAA) precursor compounds leads to increasing levels of mobile short-chain PFAS.^8^ Commonly, perfluoroalkyl carboxylic acids (PFCA, C_*n*_F_2*n*+1_COOH) and perfluoroalkyl sulfonic acids (PFSA, C_*n*_F_2*n*+1_SO_3_H) are considered short-chained for carbon chain lengths (*n*) below seven and six, respectively.^9^ For the two most well-known PFAS, perfluorooctanoic acid (PFOA) and perfluorooctane sulfonic acid (PFOS), adverse health effects have been described extensively.^2,9^ Among others, PFAS exposure is suspected of causing thyroid hormone system disruption, decreased immune function, and liver diseases.^10^

PFAS, particularly PFAA, are exceptionally inert toward chemical and biological degradation.^4^ Many PFAS are highly soluble in water and are thus ineffectively removed with conventional wastewater treatment technologies.^1,4^ For these reasons, reducing PFAS concentrations in contaminated water to below guideline levels^11^ has proven extremely challenging.^12,13^ To mitigate these difficulties, combining two or more technologies in a treatment train is considered a necessary approach for future PFAS mitigation.^14^ Specifically, combining an appropriate preconcentration technology with an on-site degradation technology is of interest to harvest efficiency from the degradation step.^14−17^

Foam fractionation (FF) is an example of such a preconcentration technology.^18^ Its suitability for the treatment of PFAS-contaminated water has been well described in academic literature.^19−25^ FF exploits the surfactant properties of common PFAS by adsorbing the compounds on rising air bubbles. If the surfactant concentration of the feed water is sufficiently high, PFAS can be harvested as a concentrated foam from the top of the water and treated further. The resulting de-foamed effluent has substantially reduced PFAS concentrations and can either be discharged to the environment or subjected to further treatments.^20^ The advantages of the FF technology compared to conventional preconcentration technologies, e.g., adsorption to activated carbon or membrane filtration, are its low use of consumables and its robustness against complex and variable water matrices.^20,22,25^ A disadvantage is that it only works for surface-active PFAS and is thus less efficient for the removal of short-chain or non-amphiphilic PFAS.

A promising destructive technology for the resulting PFAS-enriched foam is electrochemical oxidation (EO). Remediation companies are starting to apply the FF-EO treatment train commercially,^26^ but no systematic or modeling investigations have been described in academic literature yet. EO using anodes with a high O_2_ evolution overpotential has been successfully applied for the degradation of inert organic water pollutants.^27^ Specifically, boron-doped diamond (BDD) electrodes are often the material of choice for their excellent mechanical, chemical, and thermal stability, as well as their high electron transfer ability.^28^ Effective PFAS degradation on BDD electrodes has been demonstrated on the laboratory scale.^29,30^

EO studies commonly use artificially increased PFAS concentrations in synthetic solutions and thereby likely overestimate the treatment efficiency due to negligible matrix effects.^31^ In order to reach high PFAS degradation in environmental matrices, it is necessary to increase the total energy density by increasing either the total current or the total time. Otherwise, the presence of organic matter, scavenging compounds, and inorganic salts prevents efficient treatment.^32^ Differences in treatment effectiveness upon switching from artificial to natural matrices have been extensively shown,^30,32−35^ with decreased efficiencies especially noticeable for long-chain compounds^30^ and at high chemical oxygen demand (COD) concentrations under current limiting conditions.^32^

Mass transfer limitations also complicate the large-scale application of electrochemical PFAS degradation. To minimize operational costs and maximize energy efficiency, it is important to remain in the reaction-limited operational regime.^27^ Scale formation on electrode surfaces prevents the migration of PFAS to the electrode surface, thereby creating a mass transfer limitation that needs to be removed by acid rinsing.^36^ Similarly, fluorination of the BDD surface after PFAS degradation causes lower degradation rates but can be reversed by UV irradiation.^37,38^ Mass transfer limitations can also be reduced by keeping initial PFAS concentrations high^39^ or by using innovative turbulence-enhancing reactor designs.^40,41^ Finally, decreasing the current density over time can help to remain in the reaction-limited regime and thereby provide energy-efficient degradation at the cost of higher treatment times.^42^

The formation of toxic degradation byproducts forms a substantial obstacle for industrial applications of the electrochemical technology. If the total applied energy density is not sufficient, PFAS are merely degraded to shorter-chain compounds.^30^ Additionally, in water containing chloride or bromide at relevant concentrations, the formation of perchlorate, bromate, and toxic organic halides is a concern.^35,41^ It is therefore important to evaluate the toxicity of the electrochemically treated water. This assessment can be done using effect-based bioassays, as exemplified by other studies.^43−46^

The exact mechanism of PFAS degradation on BDD electrodes is still under discussion. However, there is a consensus that an initial direct electron transfer to the BDD surface is rate-limiting in the degradation of PFOA and PFOS.^31,40^ This hypothesis is supported by PFAA being insusceptible to degradation by direct hydroxyl radical oxidation.^47^ Moreover, the addition of a radical scavenger was shown not to affect PFAS removal rates by EO on BDD electrodes.^33^ A proposed mechanism for the electro-oxidation of PFAS shows that secondary radicals are involved in the degradation after the rate-limiting step.^31^ The presence of radical scavengers at high concentrations may therefore still prevent effective PFAS degradation in complex matrices.

Most studies conclude that PFOS oxidation follows the same pathway as PFOA oxidation. An initial electron transfer from the sulfate group to the anode leads to the formation of short-chain PFCA as intermediate degradation products, without the formation of any short-chain PFSA. However, in certain studies, the formation of perfluorobutane sulfonate (PFBS),^41,48^ perfluorohexane sulfonate (PFHxS),^41^ and perfluoroheptane sulfonate (PFHpS)^32^ has been observed. An alternative degradation mechanism, in which PFOS degradation can lead to both PFCA and PFSA formation, is given in Pierpaoli et al. (2021).^32^ Nonetheless, numerous studies, wherein increasing short-chain PFSA concentrations were not measured, exist as well.^29,34,36^

Here, we improve the understanding and demonstrate the high-technology readiness level of an FF–EO treatment train for the remediation of PFAS-contaminated groundwater and landfill leachate. Pilot-scale experiments were performed with real water matrices that were comprehensively analyzed for their general chemistry characteristics. EO was applied directly to the groundwater and leachate, as well as to collapsed foam produced in an on-site FF process from both water types. The objectives were to (i) evaluate the treatment effectiveness extensively using target and non-target methods, such as the total oxidizable precursor (TOP) assay and extractable organofluorine (EOF) analysis, (ii) assess the toxic potency of the water before and after treatment using two in vitro bioassays, and (iii) develop an extensive numerical model incorporating the coupled mass balances of 10 PFAS, enabling theoretical insights into the electrochemical degradation kinetics. The findings delineate both the potential of the FF-EO treatment train and limitations that need to be overcome to achieve industrial-scale on-site destructive PFAS treatment.

## Materials and Methods

### Treatment Pilot

The treatment was carried out on-site at the Hovgården landfill in Uppsala, Sweden, which has been the subject of previous studies regarding PFAS treatment.^19,22^ Leachate was collected directly from the influent to the on-site leachate water treatment plant, and groundwater was extracted from an observation well downgradient of the landfill from approximately an 8 m depth below ground surface and 7 m depth below the groundwater table. High-density polyethylene bottles were rinsed three times with methanol (LC-grade, Merck, Germany) prior to their use for sampling.

### Foam Fractionation

**Figure 1:**
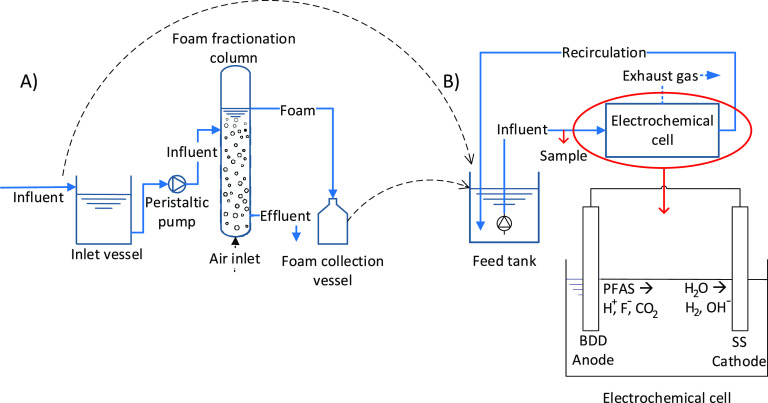
Schematic overview of (A) the FF (adapted from Smith et al. 2022)^22^ and (B) the EO process. The FF was a continuous process, but the EO experiments were done batch-wise. During the EO experiments with fractionated foam, the exhaust gas outlet shown in (B) was closed, i.e., all gas and water was recirculated to the feed tank. Electrochemical degradation experiments were done with groundwater and leachate, as well as with foam produced from both water types, all in separate duplicate experiments. The influent to the FF (A) was groundwater or leachate. The influent to the EO (B) was groundwater, leachate, or fractionated foam produced from either groundwater or leachate.

A previously described and optimized FF setup was used for the production of foam from groundwater and leachate for the EO.^22^ This system was run in continuous mode using a 19 cm diameter column with the liquid level at 1.63 m. The residence time, air flow rate, and collected foam fraction were 20 min, 10 L min^–1^, and 10%, respectively. The foam fraction is the percentage of influent water that was taken from the reactor as foam. A schematic overview of the test setup is given in Figure 1A. The system was left to run continuously for 9 h, with 250 mL of influent and effluent and 100 mL of foam collected after 2, 4, 6, and 8 h for PFAS analysis. Additionally, influent and effluent samples for organofluorine and bioassays (750 mL) and TOP assay analysis (200 mL) were collected after 2 and 6 h, and general chemistry samples of the influent and the effluent (1 L) were collected after 5 h.

### Electrochemical Oxidation

Groundwater and leachate were subjected to EO treatment in separate batch tests at 50 and 150 L total volume. The produced foam from both water types was only tested at a volume of 50 L. For each of these experiments, 9 h batch tests were carried out in duplicate, using a 20 L flow-through cell with a total active BDD anodic and stainless-steel (SS; grade 304) cathodic surface area of 9 200 cm^2^ each. The BDD electrodes were manufactured by Nova Diamant AB, had niobium as the base material, and were coated on both sides. Individual SS and BDD electrodes had a shape of 5 × 20 cm (total area of 200 cm^2^ per electrode) and were mounted alternatingly with a spacing of 3 mm. 23 BDD and 24 SS electrodes were stacked in a package, giving an active area of 4600 cm^2^ for each (the outside area of the two outermost SS electrodes was not considered part of the active electrode area). Two of these packages were used in series in the flow-through cell, with a diffusor between them to reduce mass flow limitations.

Power was supplied to this cell at a constant current of 231 A using an Agilent Technologies power supply (N8722A). The effluent from the flow-through cell was recycled to the inlet cell through steel spiral-reinforced PVC hoses with a 40 mm inner diameter at a flow rate of approximately 12 L min^–1^. A schematic overview of the test setup is given in Figure 1B. For the tests with groundwater at 150 L, a portable stirrer (KGC, 1100W) was used to mix the inlet tank. In between each test, the system was rinsed with approximately 20 L ∼2.5 g L^–1^ (pH 2–3) of citric acid solution.

250 mL samples for target PFAS analysis were collected from a valve before the electrochemical cell at times 0, 0.5, 1, 2, 3, 4, 5, 7, and 9 h for tests with leachate and groundwater and at 0, 1, 2, 3, 4, 5, 6, 7, and 9 h for tests with fractionated foam. The pH and temperature of these samples were measured with a Hach pH meter (HQ40D multimeter with Intellical PHC101 electrode), and the voltage was read from the power supply. These results are given in Supporting Information Section 1. Additionally, samples for general water chemistry analysis (1 L), EOF, and bioassays (750 mL) and TOP assays (125 mL) were collected from the same valve at times 0 and 9 h, further referred to as, respectively, the “influent” and “effluent”.

For the EO on the fractionated foam, the formation of foam in the gas outlet prevented an effective electrochemical treatment. Hence, this outlet was closed, and all the exhaust gas exited the reactor through the same recirculation hose as the water during these experiments. Foam formation occurred in the feed tank during the start of each EO experiment, but this had always mostly disappeared by the first sampling occasion. To assess the loss of PFAS in aerosols exiting the inlet tank, two stacked pre-combusted quartz microfiber filters (⌀ 11 cm, QM-A, Whatman) were placed in an aluminum holder (Tisch Environmental) on top of the inlet tank during each of the runs with foam. These filters have been used previously for sampling PFAS in aerosols with a size range between 0.1 and 2 μm,^49^ see also Supporting Information Section 3. The system was otherwise entirely airtight, so all air exiting the system passed through these filters.

### Analyses

#### General Water Chemistry

Selected water samples were sent to ALS Scandinavia, Danderyd, Stockholm, for the analysis of inorganic elements, dissolved organic carbon (DOC), total organic carbon (TOC), COD, nutrients, turbidity, conductivity, alkalinity, and pH. More details and results are presented in Supporting Information Section 2.

#### Target PFAS and TOP Assays

All target PFAS analyses (*n* = 29, full names of all PFAS compounds are given in Table S3) on groundwater, leachate, and collapsed foam for the electrochemical treatment were done in analytical duplicates, so each 250 mL sample was split in two samples of 125 mL, i.e., *n* = 4 for all time points (analytical duplicates + experimental duplicates). The influent, effluent, and collapsed foam samples (*n* = 4 for each) from the FF were analyzed without analytical duplicates. The analytical methods for both the water samples and quartz microfiber filters are described in Supporting Information Sections 3 and 4 and have also been described in detail previously.^22,49^ The influent and effluent samples of all electrochemical and FF tests were also analyzed with TOP assays, which aim to oxidize unknown PFAA precursors to PFCA to enable concentration measurements with targeted analysis. The TOP assay method is described in Supporting Information Section 5 and has been previously described by Houtz and Sedlak (2012).^50^

#### Extractable Organofluorine

EOF is a measurement of the entire organofluorine content of an extract, without giving any information on the molecular structure of individual organofluorine compounds. 750 mL of influent and effluent water from each treatment experiment were filtered and extracted using the same method as for the target PFAS analysis. Because these extracts were also used for bioassay analysis, extra rinsing steps that are normally included in extractions for EOF to remove fluoride ions^51^ were omitted. Extracts were concentrated to 200 μL in methanol, i.e., the concentration factor was 3750. Measurements of EOF were carried out using a Thermo-Mitsubishi combustion ion chromatograph and a previously developed method,^52^ see also Supporting Information Section 6. Extracts of the foam influent to the EO were diluted twice prior to EOF analysis.

#### Bioassays

To assess the effect of the evaluated PFAS treatment technologies on the toxic potency of the groundwater and leachate, two bioassays were carried out on the undiluted extracts from the EOF analysis. First, the transthyretin (TTR)-binding assay was used to assess the thyroid toxicity of the PFAS-contaminated water. TTR is a distributor protein that binds the TH-precursor thyroxine (T_4_) and transports it to target tissues. PFAS can compete with T_4_ for the binding to TTR, thereby preventing effective transport of TH.^44,53^ Additionally, the more generic *Aliivibrio fischeri* bioluminescence assay was carried out to give information on the general toxic potency of the water.^43,54^ Herein, exposure of the marine bioluminescent bacterium *A. fischeri* to toxic components in the extracts results in a decrease in bioluminescence compared to a blank caused by the inhibition of the bacterial metabolism.^43^ This type of bioassay has been used previously to evaluate the effectiveness of water treatment with advanced oxidation.^45^ Assay responses were expressed as PFOS-equivalent and triclosan-equivalent concentrations, respectively, and for the TTR assay also, the expected PFOS-equivalent concentration based on the measured target PFAS concentrations were calculated. Detailed methods for both bioassays, together with quality control data, are given in Supporting Information Section 7.

### Data Treatment

All data analysis and plotting were done in MATLAB (R2020b). The treatment efficiency (*E*) was calculated as per eq 1, with *C*_Ef_ and *C*_In_ being the effluent and influent concentrations of the corresponding treatment process, respectively. The degradation efficiency of the entire treatment train (*E*_tt_, Figure 6) was calculated as per eq 2, with *C*_Ef,FF_ and *C*_In,FF_ the effluent and influent concentrations from the FF, respectively, and *C*_Ef,EO on foam_ being the effluent concentration of the EO on the foam produced in the FF. The factors 0.9 and 0.1 come from the fact that 90% of the FF influent exits the column as effluent and 10% as foam. The foam is subsequently electrochemically degraded to form EO effluents. Minimum efficiencies were determined based on the maximum effluent concentration and the minimum influent concentration, and vice versa for the maximum efficiencies.
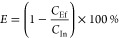
1
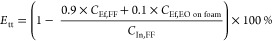
2

### PFAS Degradation Kinetics Model

A coupled ordinary differential equation (ODE) model to describe the degradation and formation of PFAS in an electrochemical flow cell was developed as a discretized 2D model along the axial position of the reactor. The model was developed using MATLAB (R2020b). It builds on a previous model for the degradation of PFOA developed by Urtiaga et al.,^39^ but the current model removes the assumption that the variation with time of the PFOA concentration is negligible compared to its variation along the axial position of the reactor (i.e., ). Moreover, it coupled the degradation and formation of a total of 10 PFAS rather than only PFOA.

All PFCA and PFSA with perfluorocarbon chain lengths between four and eight were included in the model, which together made up over 95% of the influent total target PFAS concentration in all electrochemical experiments. The model couples all degradation reactions to each other by incorporating the co-occurring stepwise degradation mechanism to shorten perfluorinated chain lengths.^31,32^ Hence, the degradation rate of the PFCA with a chain length of *n* is included as a formation rate of the PFCA with chain length *n* – 1. As explained in the introduction, the situation is more complex for PFSA, which can degrade to both PFCA and PFSA. For simplicity, PFOS and PFHpS were assumed to degrade only to PFCA since the clear formation of PFHpS or PFHxS was not observed. Contrarily, PFHxS and PFPeS were assumed to degrade only to PFSA. PFBS was assumed to degrade only to PFBA, which has been confirmed in at least two previous studies.^29,55^ Degradation of precursors to PFOA and PFOS was included as well, but since the TOP assays did not indicate the presence of any PFAA precursors, PFOA and PFOS precursor concentrations and rate constants were set to zero. For the same reason, precursor degradation to any other PFAA was also not included.

A detailed description of the model equations is given in Supporting Information Section 8. In brief, for each compound, an ODE in the form of eq 3 was derived, combining all formation and degradation reactions as functions of axial position and time. Here, *Q* is the flow rate (m^3^ min^–1^), *A*_cell_ is the cross-sectional area of the reactor (m^2^), *C*_*i*,*n*_ is the concentration of compound *i* at axial position *n* (M), *z*_*n*_ the axial position in the reactor at node *n* (m), and *k*_*i*_ the degradation rate constant of compound *i* (min^–1^). The sum term includes all reactions that lead to the formation of compound *i*. This set of equations was solved using the MATLAB ode23 solver, which is a built-in software function.
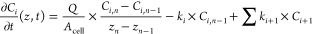
3

The values for the kinetic constants (Table S7) were found by minimizing the sum of squared errors between the model and experimental results from the 50 L groundwater tests. This calibration was done sequentially, in the order PFOS < PFHpS < PFHxS < PFPeS < PFBS < PFOA < PFHpA < PFHxA < PFPeA < PFBA, since the model results for shorter chain compounds depend on the degradation rates of long-chain compounds but not vice versa. To verify the model performance, the modeled degradation at a volume of 150 L using these same calibrated constants was then compared to the experimental results of the 150 L groundwater experiment. For simplicity, the degradation rate constants were assumed to be independent of concentration changes of any matrix compounds, such as TOC or chloride that may co-occur with PFAS degradation. Despite slight differences between the groundwater and leachate matrices (Tables S1 and S2), the constants calibrated using the 50 L groundwater results were able to predict the PFAS degradation in leachate reasonably accurately (as presented below). For the results of the experiments with fractionated foam, separate kinetic constants were calibrated.

## Results and Discussion

Mean Σ_29_PFAS concentrations and the corresponding standard deviations prior to any treatment (*n* = 12) were 3.1 ± 0.4 μg L^–1^ in the groundwater and 2.2 ± 0.2 μg L^–1^ in the landfill leachate. The groundwater and leachate, respectively, had mean DOC concentrations of 34 and 43 mg L^–1^, nitrate concentrations of 0.3 and 41 mg L^–1^, iron concentrations of 2.1 and 2.7 mg L^–1^, a pH of 7.5 and 7.8, and a conductivity of 450 and 470 mS m^–1^. Detailed results of how each treatment step affected the general water chemistry are given in Supporting Information Section 2. Mean PFAS concentrations in the raw waters and after each treatment step are given in Tables S8 and S9.

### Foam Fractionation

**Figure 2:**
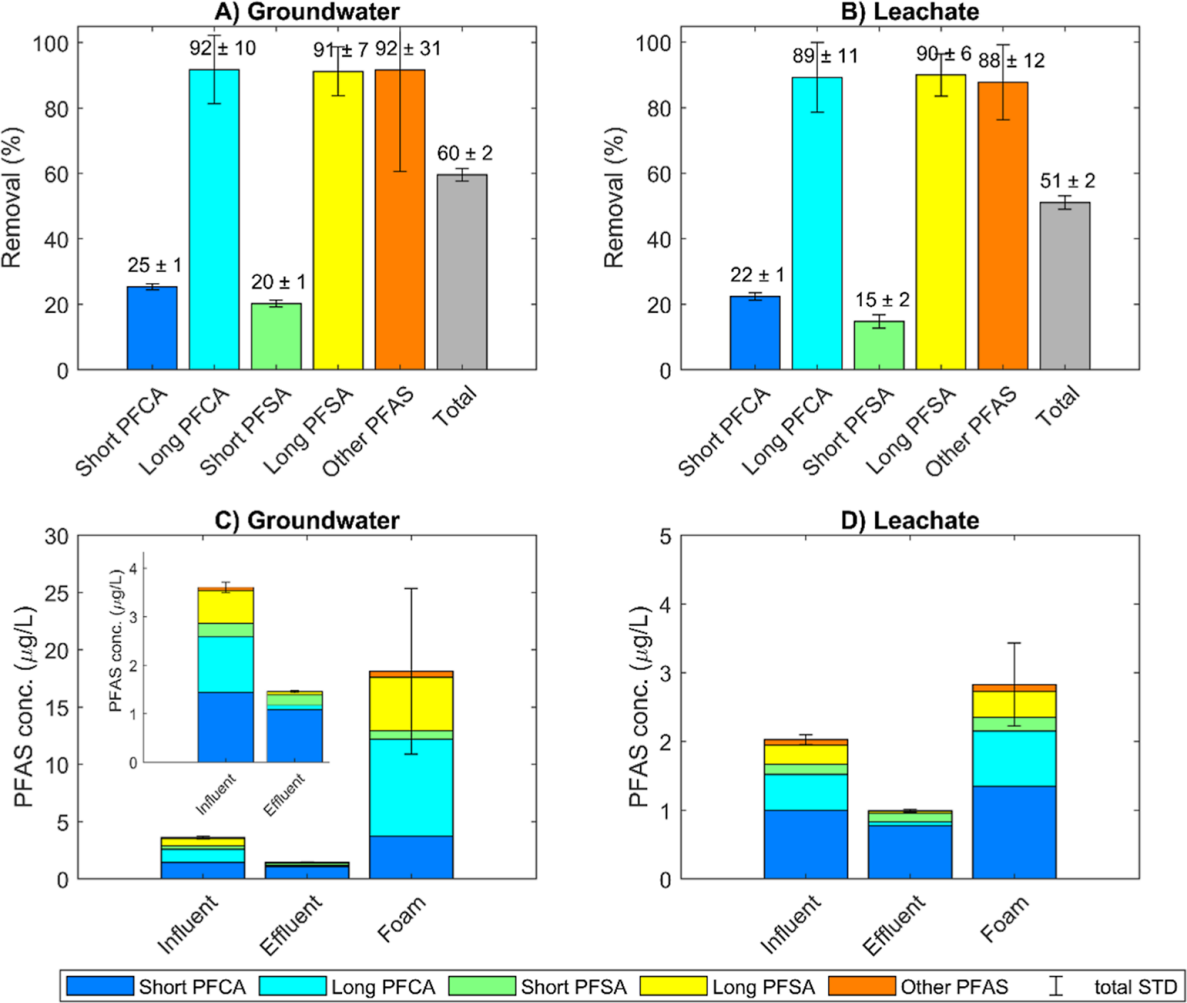
FF treatment effectiveness in terms of long-chain and short-chain PFAS removal [(A,B), %] and PFAS concentration [(C,D), μg L^–1^] for groundwater (A,C) and leachate (B,D) before treatment and in the foam. Error bars represent the total standard deviation over the four samples taken during the FF treatment (*n* = 4). The foam concentrations also include the samples (*n* = 4) taken from the bulk foam prior to EO (i.e., total *n* = 8 for the foam). The insert in (C) shows the influent and effluent concentrations of the groundwater in more detail.

The mean ΣPFAS removal effectiveness for the FF treatment was 60% in the groundwater and 51% in the leachate. Because of their higher adsorption coefficients to the air–water interface, long-chain PFAS were removed better than short-chain PFAS. This is a well-known limitation of the FF technology for PFAS removal.^19−22,25^ The higher ΣPFAS removal in the groundwater is caused by this same limitation since the groundwater contained a higher percentage of long-chain PFAA (50%) than the leachate (40%, Figures 2C and 1D). As illustrated in Figure 2A,B, the removal of these long-chain PFAA reached approximately 90% in both water types.

Average recoveries of the influent ΣPFAS in the effluent and the foam were 87 ± 35% and 58 ± 13% for groundwater and leachate, respectively. Explanations for the loss of PFAS from the mass balance have been investigated and discussed in previous work and are likely to include sorption to reactor walls and emissions to air.^19,22,56^ For the system used in this study, the high variability in mass balance closure mostly originated from the highly variable foam concentrations, while the effluent concentrations were relatively constant (Figure 2C,D). The low ΣPFAS recovery for the leachate indicates that not all PFAS that were removed from the influent were captured in the foam, which would imply that these PFAS were not degraded in the EO. Conversely, the mass balance for the groundwater closed considerably better. Mass balance closures for commercial FF systems have not yet been reported,^20,25^ which is an important knowledge gap for this treatment technology.

### Electrochemical Oxidation

**Figure 3:**
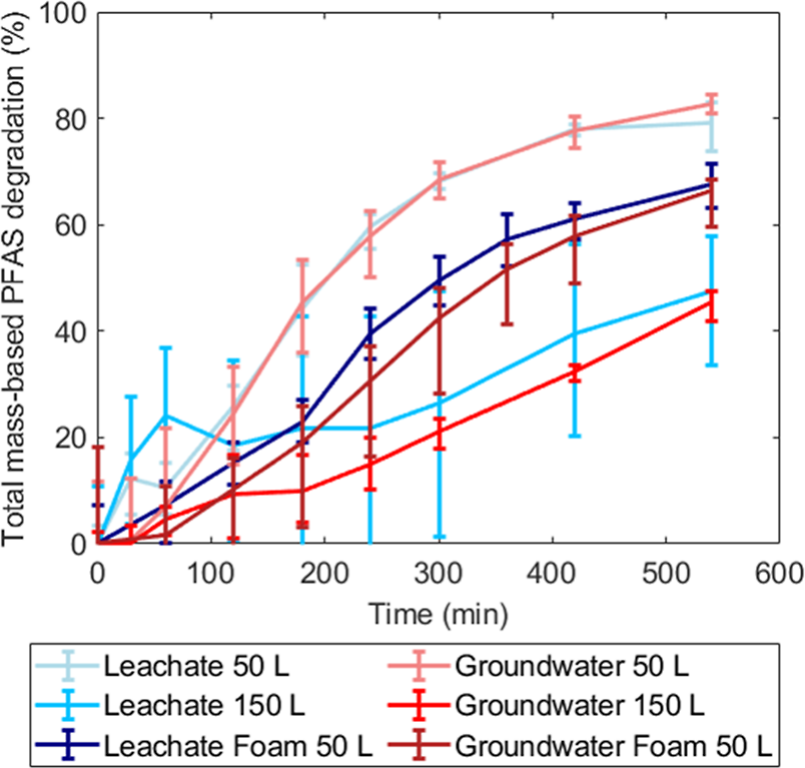
ΣPFAS degradation over time in all electrochemical experiments. Error bars represent min and max values based on the experimental and analytical duplicates (i.e., *n* = 4); lines connect the means.

Up to 84% ΣPFAS degradation was achieved after 540 min of EO (Figure 3). Unlike for the FF, the EO treatment effectiveness was similar for groundwater and leachate. For the experiments at 150 L volume, the recirculation was not sufficient to keep the inlet tank well-mixed, causing a high variability within the leachate results. A stirrer was installed for the groundwater tests at 150 L to prevent this issue, leading to more reproducible results. As expected,^32,42^ the degradation was highly dependent on the specific charge *Q* (A h L^–1^, eq S3). Because of the inverse relation between *Q* and treated volume, the PFAS degradation in both matrices remained lower at a higher volume. However, when plotted against the specific charge instead of time, the results were independent of volume, as illustrated in Figure S5.

A lower final degradation effectiveness of approximately 65% was achieved for the fractionated foam of both matrices, as could be expected based on the higher initial PFAS concentrations in the foam. ΣPFAS concentrations were 2.2 and 2.8 μg L^–1^ in the leachate and groundwater, respectively, versus 3.6 and 19 μg L^–1^ in the corresponding concentrated foams. The higher PFAS concentrations may have accelerated diffusive mass transfer from the bulk foam to the electrodes. Consequently, electron transfer at the BDD anodes became rate-limiting rather than mass transfer, making the degradation curve more linear^40^ (Figures 3, S6 and S7). Conversely, the degradation in the unfractionated waters was still limited by mass transfer and could thus be made more energy-efficient by implementing turbulence-inducing reactor designs.^40,41^ Additionally, electrode scaling (Tables S1 and S2) or fluorination may have contributed to lower degradation rates in the foam experiments.

**Figure 4:**
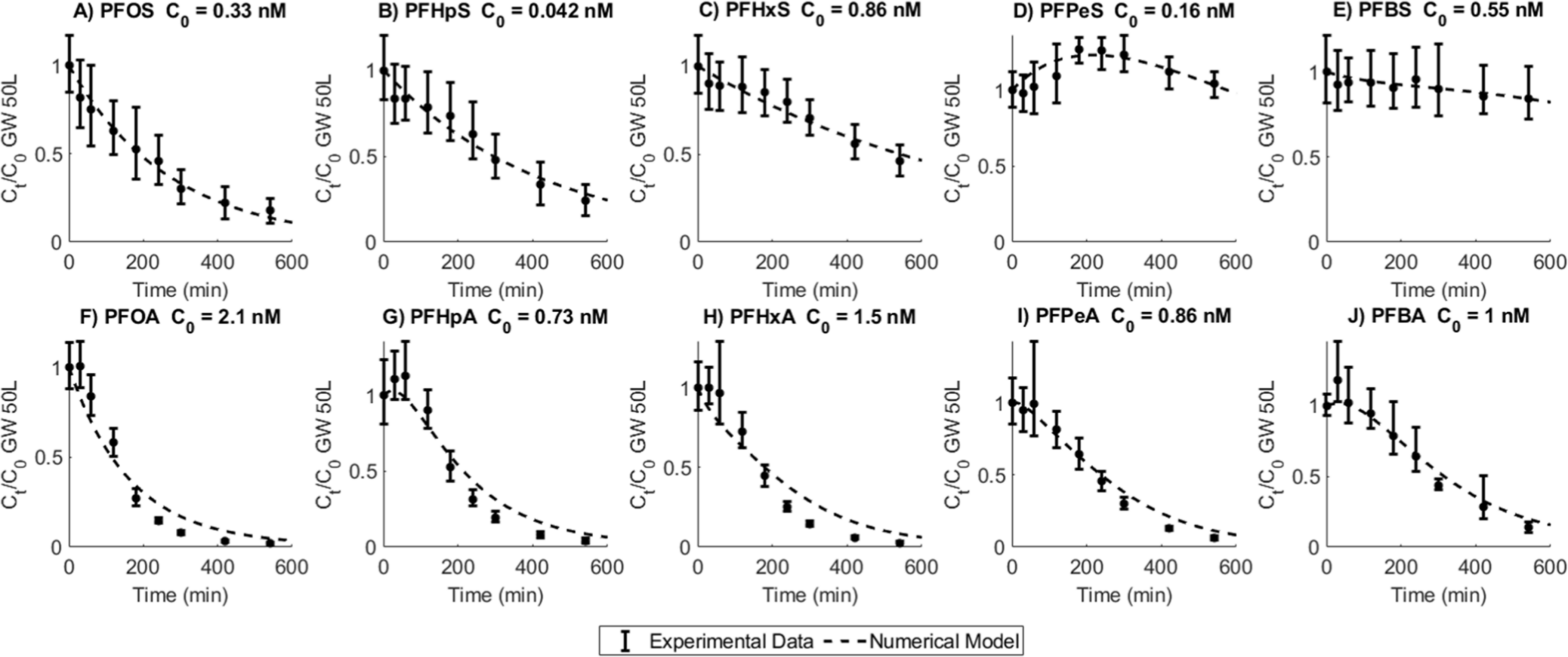
Individual degradation of PFAA with chain lengths up to 8 for the EO run with 50 L groundwater. The initial concentration of each PFAA is stated in the heading, and shorter-chain PFAS could be formed from the degradation of longer-chain PFAS. Error bars represent min and max values based on the experimental and analytical duplicates (i.e., *n* = 4); dots represent the mean, and the dotted line is the model prediction with calibrated kinetic constants, see Table S7.

Figure 4 shows the degradation of 10 individual PFAS during the experiments with groundwater at a 50 L volume, together with the kinetic model results at calibrated rate constants (Table S7). For PFSA, the final degradation decreased in the order of PFOS > PFHpS > PFHxS > PFBS > PFPeS, whereas the numerical rate constants decreased in the order of PFPeS > PFOS > PFHpS > PFBS > PFHxS. For PFCA, the final degradation decreased as PFOA > PFHxA > PFHpA > PFPeA > PFBA and the rate constants as PFPeA > PFBA > PFHpA > PFHxA > PFOA. Since short-chain PFAS were formed as degradation products, net short-chain degradation was lower than long-chain degradation, despite higher rate constants for certain short-chain compounds. The optimization of EO-based mineralization will need to account for co-occurring short-chain PFAS formation and degradation. Earlier studies on the degradation of isolated PFAS found increased rate constants for higher chain lengths.^29,57^ The higher hydrophobicity of long-chain compounds can lead to their easier adsorption onto the BDD anode, causing faster degradation. Conversely, in the current study, the unequal initial PFAS concentrations and matrix competition effects probably contributed to the observed trend in degradation rates.

The degradation of PFSA was slower than that of PFCA, both in terms of rate and final degradation, as reported previously.^29,31,33^ This difference is attributable to the slower electron transfer from PFSA to BDD than from PFCA due to the higher electrophilicity and standard reduction potential of a sulfonic group as compared to a carboxyl group.^29,31^ Additionally, PFSA adsorb more readily to suspended solids than PFCA,^58^ which may have decreased the availability of PFSA for degradation on the BDD surface. Similar trends in the degradation were found for the remaining electrochemical experiments, with experimental and model results given in Figures S6–S10.

As visualized in Figures 4 and S6–S10, the PFAS degradation kinetics model was able to represent the EO results for leachate and groundwater well after calibration of the rate constants (Table S7). The 50 L groundwater experiments were used for this calibration, after which the model was able to adequately reproduce the degradation in leachate and groundwater at all volumes tested. A major benefit of this coupled numerical model is the capability of simultaneously accounting for formation and degradation rates of diverse PFAS, thereby eliminating the need to test individual compounds in isolated tests. The model is easily adaptable for other reactor dimensions and may be used to predict the degradation at varying treatment times or volumes.

The model, however, overestimated the PFAS degradation in the fractionated foam considerably (Figures S9 and S10). Pseudo-first-order degradation rate constants depend on matrix interferences and mass transfer limitations, in addition to intrinsic molecular properties.^31^ Therefore, the calibrated constants are likely to be different for different water types. Since the groundwater foam had higher DOC and TOC concentrations than the original groundwater (Table S1), and the leachate foam also had different concentrations of certain ions than the original leachate (particularly iron and nitrogen species, Table S2), competition from co-solutes or the presence of radical scavengers may have affected the PFAS degradation rate constants.^32^ Additionally, the high initial long-chain PFSA concentrations made the model assumptions regarding PFSA degradation pathways more influential. Implementing the wrong degradation pathway of a long-chain PFSA will have substantial effects on the modeled concentration of shorter PFAS. When calibrating the rate constants specifically for the fractionated foam, better fits were obtained (Figures S11 and S12), as discussed in detail in Supporting Information Section 8.

Figures S11 and S12 also show that PFSA degradation in the fractionated foams was very low. It is currently unclear what caused this low degradation efficiency, but it is nonetheless an important result. If EO would be used commercially for the degradation of fractionated foam, it is crucial that PFSA are degraded to a similar extent as PFCA. Particularly, PFOS is commonly included in regulations that stipulate maximum allowed concentrations in water,^1^ and PFOS had a degradation efficiency of 0% in the leachate foam and only 36% in the groundwater foam in our study.

The estimated ΣPFAS concentrations in the gas exiting the electrochemical reactor during the degradation of groundwater and leachate foam were 5.0 and 1.7 μg m^–3^, respectively. These concentrations are orders of magnitude higher than typical atmospheric PFAS levels,^59−61^ stressing the need to install appropriate air filters on exhaust gas pipes from electrochemical treatment facilities. Alternatively, where possible, the air exhaust could be coupled to the FF unit to recirculate until degradation. This loss of PFAS corresponds to only ∼1% (leachate) and ∼0.6% (groundwater) of the influent foam concentrations, which is negligible compared to the measured degradation. Concentrations in the top aerosol filters were consistently lower than in the bottom filters (Figure S2), which indicates that the escape of PFAS through both filters was probably low. Calculation methods and complete results from the aerosol analysis are given in Supporting Information Section 2.

### TOP Assay and EOF Analysis

PFCA concentrations did not increase after the TOP assay in groundwater and leachate compared to the target PFAS concentrations, indicating that oxidizable PFAA precursor concentrations were negligible.^50^ This may be because all oxidizable PFAA precursors were already degraded in the landfill. Higher EOF concentrations than explained by the target PFAS concentrations were found in most samples, indicating that unknown PFAS may be present. Nevertheless, the decrease in EOF after EO was similar to the target PFAS degradation, as visualized in Figure S3. In the FF treatment, however, EOF removal was lower than the target PFAS removal, indicating the presence of non-standard PFAS that were not removed effectively. Further analytical work would be needed to identify these potential PFAS. With this one exception, the results of the TOP assays and EOF analysis are largely consistent in demonstrating the effectiveness of the treatment (see detailed results in Supporting Information Sections 5 and 6).

It is common practice that additional washing steps are included in the extraction procedure for EOF analysis to remove fluoride, with the drawback of increasing the loss of more polar and shorter PFAS.^51^ Therefore, overall EOF recoveries might be lower in post-treatment samples, where the proportion of short-chain PFAS was higher than in pre-treatment samples. In this study, these extra washing steps were omitted, achieving nonetheless a good fluoride removal (>99%) and good overall EOF recoveries (70%) in quality control samples (which included short-chain PFAS, see Supporting Information Section 6). Accordingly, the possible overestimation of EOF removal due to PFAS recovery variability as a function of chain length is probably low.

Electrochemical treatment may result in the transformation of precursors that are not detected by the TOP assay.^34^ Schaefer et al. (2018) found that while the TOP assay substantially underestimated the organic fluorine present in AFFF-contaminated water, this additional organic fluorine did not degrade to PFAA during either the EO or the TOP assay. Specifically, the degradation of organic fluorine compounds belonging to e.g., the AmPr-FASA (*N*-dimethyl ammonio propyl perfluoroalkyl sulfonamide) class was shown not to result in the formation of PFAA, although they were degraded during EO. Because our EOF results indicated the presence of unknown PFAS while no increased PFAA concentrations were measured in the TOP assay, it is plausible that compounds such as AmPr-FASA were present. EOF results do not give structural information, so verifying that this unknown organic fluorine did not degrade to PFAA during EO was not possible in this study. However, PFAA formation due to precursor degradation was nevertheless deemed negligible and thus left out of the model.

### Bioassays

**Figure 5:**
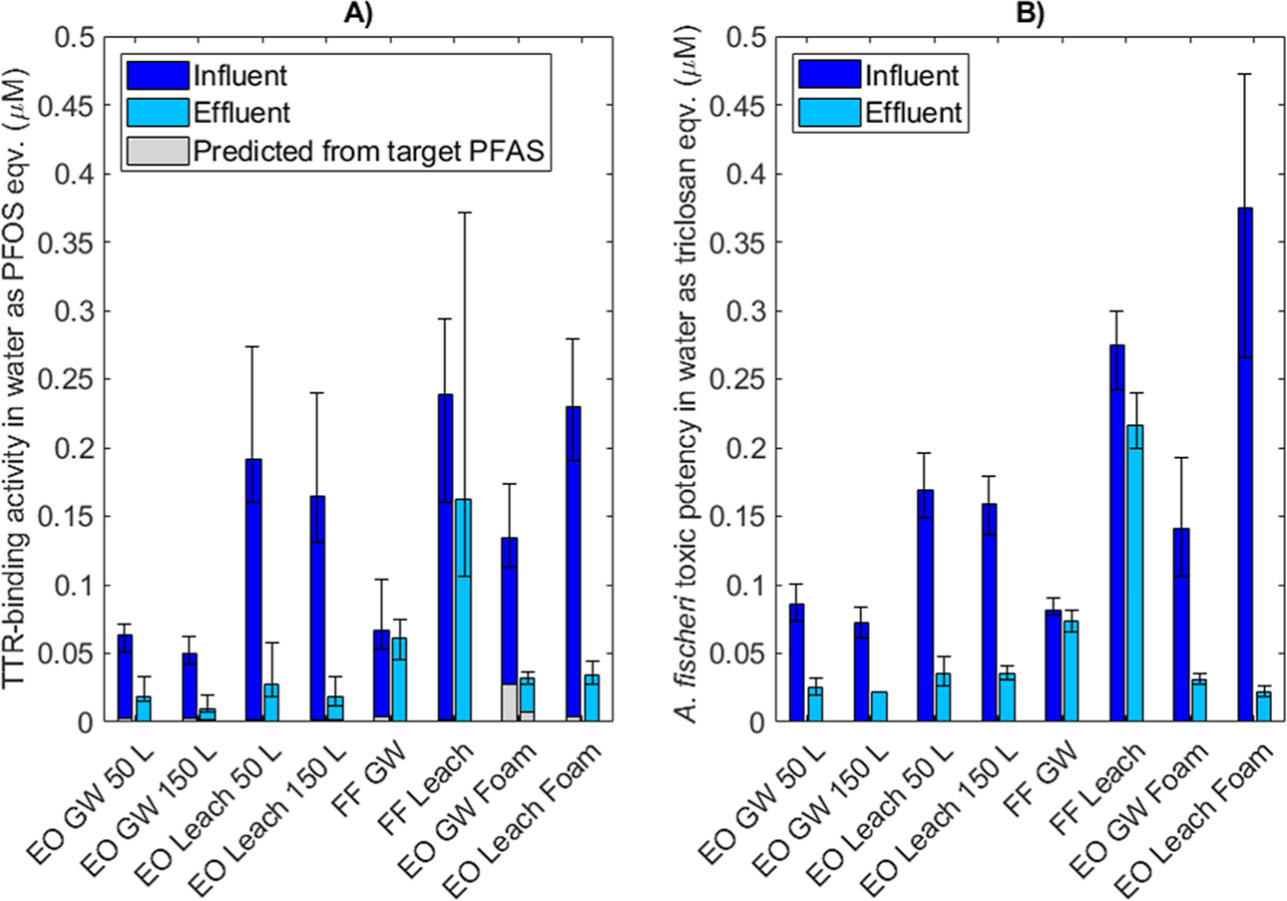
Effect of the EO and FF treatment of groundwater (GW) and leachate (leach) on (A) TTR-binding activity expressed as the mean PFOS-equivalent concentration and (B) the *A. fischeri* bioluminescence activity expressed as the mean triclosan equivalent concentration after 30 min exposure. Error bars represent min and max values based on the experimental and analytical duplicates (i.e., *n* = 4). See Supporting Information Section 7 for the calculation of the predicted TTR-binding activity of target PFAS, represented as light gray bars in (A). Significance was not calculated due to a too small independent sample size.

EO treatment resulted in a decreased capacity to compete with the thyroid hormone thyroxine (T_4_) for TTR-binding as well as a decreased effect on bacterial respiration determined with the *A. fischeri* bioluminescence assay for all experiments, as illustrated in Figure 5. Assay responses from the influent to the electrochemical effluent decreased by up to 89% (leachate 150 L) and 94% (leachate foam) for the TTR-binding and bioluminescence assay, respectively. Conversely, for the FF treatment, no major changes in TTR-binding activity and cellular toxicity from the influent to effluent were evident. Here, mean TTR-binding PFOS equivalent concentrations decreased by 9 and 32% for groundwater and leachate, respectively, and bioluminescence triclosan equivalent concentrations by 10 and 21%.

The leachate had higher activities than groundwater in both assays, despite the higher target PFAS concentrations in groundwater, which was likely due to other substances present in the leachate. The predicted fraction of the TTR-binding activity that corresponded to the measured target PFAS concentrations varied between 0.21% (FF leachate effluent) and 21% (EO groundwater foam effluent), with mean resp. median values of 5.5 and 2.4%. This implies that there were other compounds present in the extracts with TTR-binding capacity but that these compounds were also destroyed effectively in the electrochemical treatment. The effective degradation of these unidentified compounds may also explain the higher decrease in bioassay activity than in target PFAS concentrations after EO treatment on the foam (Figures 2 and 4).

### Energy Use

The energy consumption of EO can be calculated as described previously^15^ and as presented in eq S18. Since the power usage was relatively similar for all EO experiments (Figure S1B), the energy consumption was mostly dependent on the treated volume, resulting in an energy consumption after 9 h of treatment of approximately 270 kW h m^–3^ for all 50 L experiments and 93 kW h m^–3^ for all 150 L experiments. This is comparable to values calculated based on literature descriptions of other EO systems used for PFAS degradation in heavy matrices with similar efficiencies.^17,48^ Normalized energy consumptions were calculated as described by Sharma et al.^62^ and in eq S19. The normalized energy consumption per log removal of PFOA was on average 240 and 160 kW h m^–3^ for leachate and groundwater and 410 and 350 kW h m^–3^ per log removal of PFOS, respectively.

The energy consumption of a full-scale FF plant described by Burns et al.^25^ was 0.8 kW h m^–3^. Although the energy use of our pilot-scale FF system may have been somewhat higher because of the smaller scale, it was probably still negligible compared to the energy consumption of the EO. Accordingly, since the removal of both PFOA and PFOS in the FF was approximately 90%, the energy consumption to reach one log removal of PFOA or PFOS in the entire treatment train depended only on the EO system. This can be estimated as described in Supporting Information Section 9 (eq S19) and was 76 and 53 kW h m^–3^ for one log removal of PFOA from leachate and groundwater, respectively. For PFOS, the energy consumption over the entire treatment train could not be determined reliably with the current data due to its low degradation in the fractionated foam.

## Conclusions

**Figure 6:**
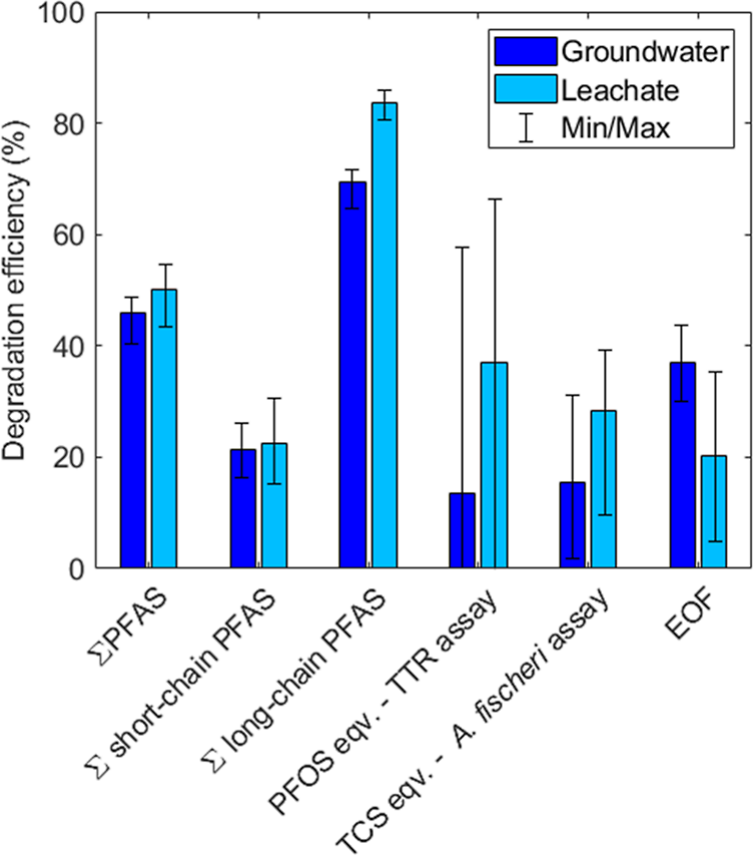
Degradation efficiency of the entire FF-EO treatment train, as defined by eq 2. Error bars represent the minimum and maximum degradation based on all respective measurements per variable, i.e., analytical and experimental duplicates for target PFAS in the EO experiments and bioassays, experimental quadruplicates for target PFAS in the FF experiments, and experimental duplicates for EOF analysis. TTR: transthyretin; TCS: triclosan; EOF: extractable organofluorine. See Supporting Information Section 2.3 for the calculation method of these efficiencies.

Figure 6 summarizes the performance of the entire treatment train in terms of target PFAS and EOF degradation as well as reduction in bioassay activity. Due to the poor removal of short-chain PFAS in the FF step combined with their formation in the EO, the overall degradation of long-chain PFAS (mean 77%) was more than three times higher than that of short-chain PFAS (mean 22%). For all tested treatment outputs included in Figure 6, the treatment resulted in a mean reduction of at least 13% (TTR assay, groundwater), implying that degradation exceeds byproduct formation in the EO. When treating all the water directly with EO, degradation efficiencies were higher (Figure 3), but at the cost of a higher energy use due to the 10-fold larger volume requiring energy-intensive destructive treatment.

Our results indicate three main options for improving this treatment train, which may motivate further studies advancing the presented technologies. First, operating the FF system to produce lower foam volumes will generate foam at higher PFAS concentrations, as well as enable longer treatment times in the EO because of the lower foam volume. A longer treatment time corresponds to a higher total energy density, with the additional benefit of improved mass transfer rates because of the higher concentrations. To further concentrate the foam, secondary and tertiary FF steps can be included, as has been exemplified previously.^20,25^ It should, however, be noted that the efficiency of the EO may decrease even further when more concentrated foam is treated, and that foaming in the EO may become an issue that prevents effective treatment. Second, the employment of auxiliary surfactants may lead to a higher removal of short-chain compounds in the FF. Finally, improving the mass transfer in the EO by employing innovative flow cell designs may increase the degradation rates and thereby enable complete degradation of short-chain as well as long-chain PFAS.

Other recommended areas for future research include following up on the low mass balance closure of the FF process and the low degradation of PFSA during EO treatment of the fractionated foam. If the proposed treatment train is implemented at full scale, it would be crucial to confirm that all PFAS that are removed from the influent in the FF end up in the foam, such that they are degraded with EO. Additionally, the low electrochemical degradation of PFSA in fractionated foam that was found in this study may challenge the applicability of EO as a destructive technology for PFAS-enriched foam. This low degradation may be due to artifacts or due to matrix-specific effects, but this should be confirmed by thoroughly testing and mechanistically characterizing EO on foam prior to the implementation of full-scale systems.

## Supporting Information Available

pH, voltage, and temperature results; general chemistry results; aerosol analysis—additional methods and results; analytical method—detailed methods and quality control results; TOP assays—additional methods and results; EOF analysis—additional methods and results; bioassays—additional methods and results; model—derivation of equations, calibrated rate constants and pseudocode; and additional EO results (PDF)
