# Supplementary material for: Electrochemical Oxidation for Treatment of PFAS in Contaminated Water and Fractionated Foam—A Pilot-Scale Study
Source: ACS ES T Water. 2023 Mar 15;3(4):1201–11. doi: 10.1021/acsestwater.2c00660 (PMC10111409; doi:10.1021/acsestwater.2c00660)
Supplement: Supplementary file 1 — ew2c00660_si_001.pdf [file ew2c00660_si_001.pdf]

Supplementary information to: Electrochemical oxidation for treatment of PFAS in contaminated water and fractionated foam - a pilot scale study

Sanne J. Smith[1], Mélanie Lauria[2], Lutz Ahrens[1], Philip McCleaf[3], Patrik Hollman[4], Sofia Bjälkefur Seroka[3], Timo Hamers[5], Hans Peter H. Arp[6,7], and Karin Wiberg[1]

[1]*Department of Aquatic Sciences and Assessment, Swedish University of Agricultural Sciences (SLU), P.O. Box 7050, SE-750 07, Uppsala, Sweden*
[3]*Uppsala Water and Waste AB, P.O. Box 1444, SE-751 44, Uppsala, Sweden*
[4]*Nova Diamant AB, Tryffelvägen 17, 75646, Uppsala, Sweden*
[2]*Department of Environmental Science, Stockholm University, Svante Arrhenius Väg 8, 10691, Stockholm, Sweden*
[5]*Amsterdam Institute for Life and Environment (A-LIFE), Vrije Universiteit Amsterdam, De Boelelaan 1085, 1081 HV, Amsterdam, the Netherlands*
[6]*Norwegian Geotechnical Institute (NGI), P.O. Box 3930, Ulleval Stadion, NO-080,6 Oslo, Norway*
[7]*Department of Chemistry, Norwegian University of Science and Technology (NTNU), NO-7491, Trondheim, Norway*

1. PH, VOLTAGE AND TEMPERATURE RESULTS

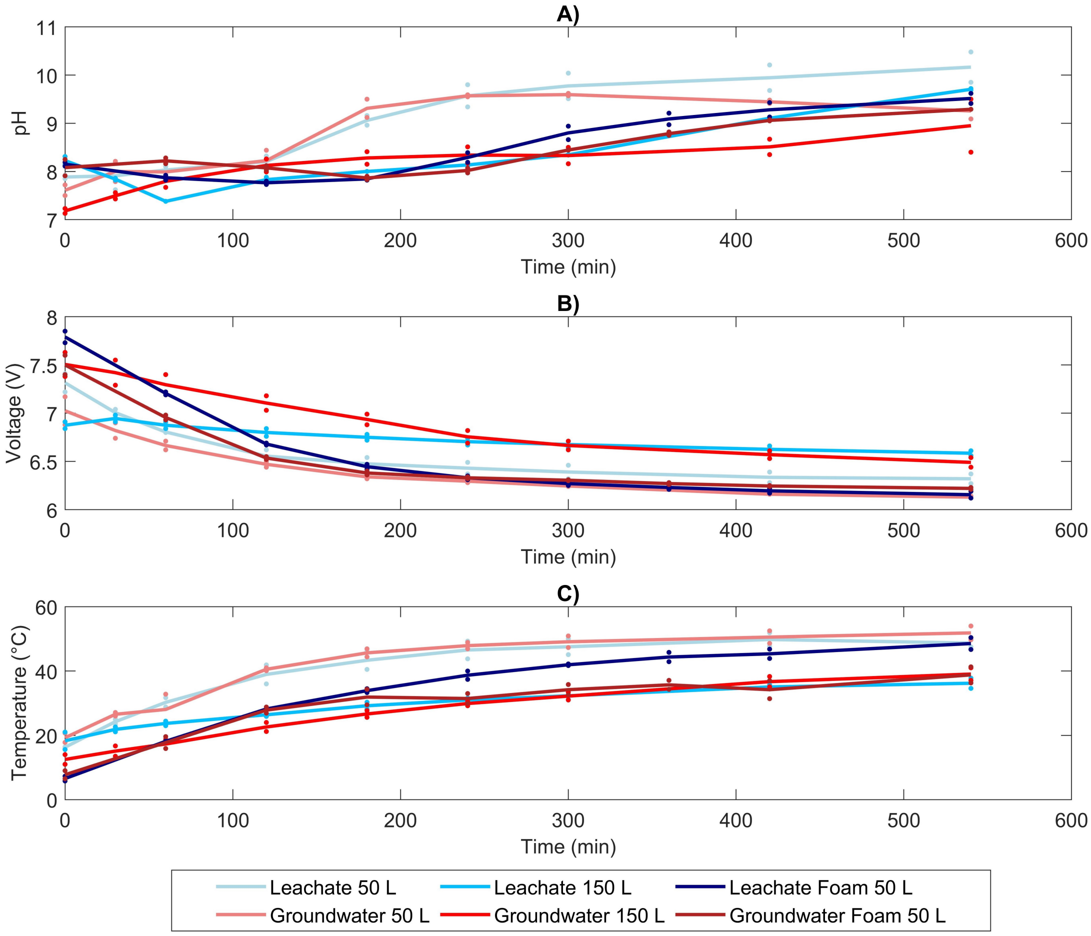

Figure S1. A) pH, B) Voltage (V) and C) Temperature (°C) over time during the electrochemical experiments. The dots represent the individual measurements for each duplicate experiment, whereas the lines connect the means of the two measurements.

2. GENERAL CHEMISTRY RESULTS

Tables S1 and S2 show the mean general chemistry of the water before and after each treatment step. The analysis of the groundwater included a few additional parameters compared to the leachate due to changes in the package offered by the analytical laboratory. Concentrations below the limit of quantification (LOQ) were set to half the LOQ. Effects of the foam fractionation (FF) treatment on the general chemistry were negligible. Conversely, the electrochemical treatment resulted in noticeably decreased dissolved organic carbon (DOC), total organic carbon

(TOC), chemical oxygen demand (COD) and dissolved solids concentrations. In this treatment, organic constituents are mostly degraded by electrochemical oxidation, whereas inorganic salts are removed by precipitation. Conversely, nitrate and to a lesser extent sulfate concentrations increased after electrochemical treatment, due to their formation from the oxidation of ammonium, nitrite and sulfite. Chloride concentrations decreased after electrochemical treatment, which indicates the formation of perchlorate or other high-valent oxidized chlorine species. These byproducts are toxic and would need to be removed in a biological post-treatment step.[1]

Table S1. Mean general chemistry of groundwater before and after each treatment step. Note the reduction of carbonate, calcium, iron, and magnesium concentrations after EO, which indicates the formation of precipitation that could lead to scaling on the electrode.

	Untreated $(n = 5)$	*FF Effluent* $(n = 1)$	*EO 50 L Effluent* $(n = 2)$	*EO 150 L Effluent* $(n = 2)$	*Foam* $(n = 2)$	*EO Foam Effluent* $(n = 2)$
Uranium ($\mu g\, L^{-1}$)	35	52	10	22	51	25
Calcium ($mg\, L^{-1}$)	230	230	19	36	200	3.0
Manganese ($\mu g\, L^{-1}$)	1000	1100	400	630	870	340
Sodium ($mg\, L^{-1}$)	680	830	630	640	850	840
Potassium ($mg\, L^{-1}$)	140	180	120	130	190	180
Iron ($mg\, L^{-1}$)	2.1	4.6	1.8	1.4	2.3	2.2
Aluminum ($\mu g\, L^{-1}$)	23	11	35	28	45	42
Copper ($\mu g\, L^{-1}$)	1000	6.0	940	1100	2700	1500
Magnesium ($mg\, L^{-1}$)	75	94	34	63	96	66
DOC ($mg\, L^{-1}$)	34	29	0.25	4.3	52	1.4
TOC ($mg\, L^{-1}$)	35	31	0.25	4.7	81	12
Phosphor ($mg\, L^{-1}$)	0.05	0.09	0.02	0.03	0.07	1.34
Nitrite ($mg\, L^{-1}$)	0.1	0.1	0.6	0.0	0.2	1.3
COD ($mg\, L^{-1}$)	25	15	0.25	0.25	31	0.25
Ammonium ($mg\, L^{-1}$)	4.7	9.7	0.21	0.03	10	0.12
Phosphate ($mg\, L^{-1}$)	0.02	0.02	0.02	0.02	0.02	0.04
Nitrate ($mg\, L^{-1}$)	0.31	0.25	6.3	6.4	0.25	8.2
Fluoride ($mg\, L^{-1}$)	0.62	0.5	0.39	0.39	0.59	0.39
Chloride ($mg\, L^{-1}$)	740	960	0.55	35	920	1.0
Sulfate ($mg\, L^{-1}$)	670	590	690	640	570	580
Turbidity (FNU)	26	72	14	16	33	15
Conductivity ($mS\, m^{-1}$)	450	550	350	350	530	440
pH	7.5	7.8	9.6	9.5	8.1	9.4
Alkalinity ($mg\, HCO_3^-\, L^{-1}$)	980	1300	58	250	1200	450

Table S2. **Mean general chemistry of landfill leachate before and after each treatment step. Note the reduction of carbonate, calcium, iron, and magnesium concentrations after EO, which indicates the formation of precipitation that could lead to scaling on the electrode.**

	Untreated $(n = 5)$	*FF Effluent* $(n = 1)$	*EO* 50 *L Effluent* $(n = 2)$	*EO* 150 *L Effluent* $(n = 2)$	*Foam* $(n = 2)$	*EO Foam Effluent* $(n = 2)$
Uranium ($\mu g\,L^{-1}$)	61	50	24	53	44	25
Calcium ($mg\,L^{-1}$)	150	150	4.4	6.3	140	2.4
Manganese ($\mu g\,L^{-1}$)	380	460	200	200	450	240
Sodium ($mg\,L^{-1}$)	640	720	660	630	700	720
Iron ($mg\,L^{-1}$)	2.7	3.7	2.0	1.8	6.8	5.2
Aluminum ($\mu g\,L^{-1}$)	42	18	34	33	79	81
Magnesium ($mg\,L^{-1}$)	61	59	30	47	56	38
DOC ($mg\,L^{-1}$)	43	44	1.8	6.9	45	1.5
TOC ($mg\,L^{-1}$)	44	44	1.5	6.8	47	1.6
Phosphor ($mg\,L^{-1}$)	0.28	0.9	0.08	0.09	0.45	0.34
Nitrite ($mg\,L^{-1}$)	11	1.0	9.1	0.12	1.1	3.7
COD ($mg\,L^{-1}$)	27	25	0.50	0.50	25	0.14
Nitrate ($mg\,L^{-1}$)	41	11	53	92	16	43
Fluoride ($mg\,L^{-1}$)	0.23	0.25	0.36	0.05	0.14	0.14
Chloride ($mg\,L^{-1}$)	900	1000	2.5	44	930	1.0
Sulfate ($mg\,L^{-1}$)	220	130	250	250	120	130
Conductivity ($mS\,m^{-1}$)	470	500	360	360	470	380
pH	7.8	7.6	11	9.6	8.1	9.8
Alkalinity ($mg\,HCO_3^-\,L^{-1}$)	1100	1200	360	400	1200	520

3. AEROSOL ANALYSIS

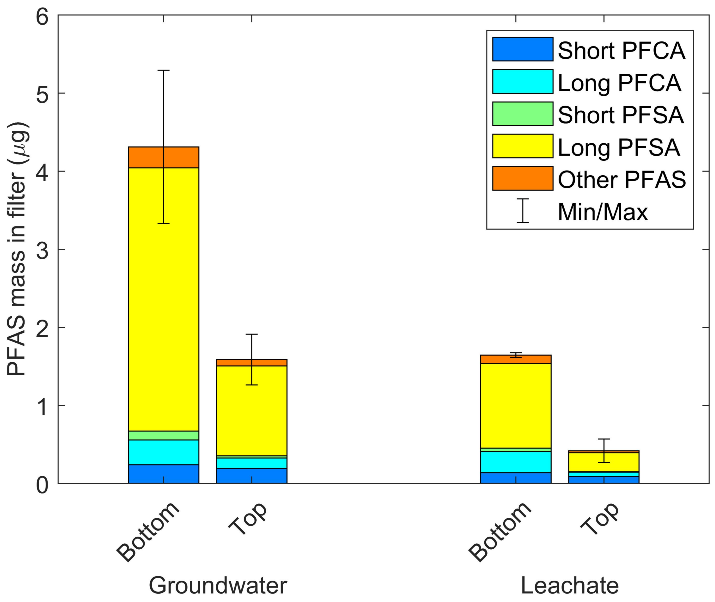

Figure S2. Measured PFAS mass in the aerosol filters placed on top of the inlet cell during the electrochemical treatment of the fractionated foam. Air exiting this tank passed through the bottom filter before passing through the top filter. Error bars represent the minimum and maximum ΣPFAS mass detected over the duplicate runs.

The quartz microfiber filters used for aerosol collection were extracted according to a modified protocol described by Casas *et al.*.[2] Each filter was transferred to a 50 mL polypropylene (PP) tube and 50 µL of an internal standard (IS) mixture containing 50 ng mL^{-1} of each individual compound was added.[3] One blank with a clean filter and one blank without filter were included as well. Then, 15 mL of methanol was added to each tube, after which they were vortexed briefly and sonicated for 20 min. The methanol was decanted into a second PP tube, and the extraction was repeated twice with 5 mL of methanol. The combined methanol fractions were concentrated to 0.5 mL under a gentle stream of N_2 (N-EVAP™112, Organomation Associates Inc., USA) and transferred to 1.5 mL Eppendorf tubes. Each PP tube was rinsed three times with methanol, which was added to the corresponding Eppendorf tube, and the extracts were concentrated to 0.5 mL under N_2 again. Finally, the Eppendorf tubes were centrifuged for 15 min at 4000 rpm (Eppendorf centrifuge 5424R) and 150 µL supernatant was transferred to an analytical PP insert vial for UPLC-MS/MS analysis according to the same protocol as for the water and foam extracts, see section 4 and Smith *et al.*, 2022.[3]

The LOQs for the determination of the aerosol mass per filter are given in Table S3. These LOQs correspond to either the instrument LOQ of 0.1 ng, or the highest mass detected in the extraction blanks. In the data analysis, values below the LOQ were set to 0, since the reported concentrations correspond to minimum concentrations. The field blank contained a ΣPFAS mass of 57 ng, the majority of which was PFOS (45 ng). This high concentration indicates that PFAS had already been emitted to the air during previous runs with the electrochemical system, which contaminated the field blank. However, as illustrated in Figure S2, the PFAS levels in the filters used during electrochemical runs were much higher than in the field blank.

Although the PFAS levels in the bottom filters were clearly higher, PFAS were detected in the top filters at high concentrations as well. Hence, PFAS passed through the bottom filter during the runs. This breakthrough could be due to saturation of the bottom filters. Alternatively, PFAS from the air surrounding the system may have adsorbed to the top filter. Nonetheless, for further calculations, we assumed that the PFAS found in both filters originated from the treatment. Since it is possible that not all PFAS in the exhaust gas was caught in the filters, calculated concentrations represent minimum values.

Faradays law, given in Equation S1, was used for the calculation of the H_2 gas formation rate ($r_{H_2}, L\,s^{-1}$) in the electrochemical system, with F Faradays constant (98465 $C\,mol^{-1}$), I the current (231 A), Z the number of electrons per H_2 molecule formed (2) and V_m the molar volume of an ideal gas (22.4 L). For simplicity, we assumed a cathodic current efficiency towards the reduction of water of 100 % and ignored the formation of gaseous byproducts at the anode. Exhaust gas PFAS concentrations were subsequently calculated as per Equation S2, with M_{tot} the total PFAS mass from both filters and t_{tot} the total treatment time (32400 s). It should be noted that this calculation is an approximation.

$$r_{H_2} = \frac{I}{F \cdot Z} \cdot V_m \tag{S1}$$

$$C_{gas} = \frac{M_{tot}}{t_{tot} \cdot r_{H_2}} \tag{S2}$$

Table S3. **LOQ in aerosol filters**

Compound	$LOQ\ (ng\ filter^{-1})$
Perfluorobutanoic acid (PFBA)	1.9
Perfluoropentanoic acid (PFPeA)	0.1
Perfluorobutane sulfonate (PFBS)	0.1
Perfluorohexanoic acid (PFHxA)	0.1
4:2 Fluorotelomer sulfonic acid (4:2 FTSA)	0.2
Hexafluoropropylene oxide dimer acid (HFPO-DA)	0.2
Perfluoropentane sulfonate (PFPeS)	0.1
Perfluoroheptanoic acid (PFHpA)	0.1
4,8-dioxa-3H-perfluorononanoic acid (NaDONA)	0.1
Perfluorohexane sulfonate (PFHxS)	0.2
Perfluorooctanoic acid (PFOA)	0.1
6:2 Fluorotelomer sulfonate (6:2 FTSA)	0.5
Perfluoroheptane sulfonate (PFHpS)	0.1
Perfluoroethyl-cyclohexane sulfonate (PFECHS)	0.1
Perfluorononanoic acid (PFNA)	0.1
Perfluorooctane sulfonamide (FOSA)	0.2
Perfluorooctane sulfonate (PFOS)	4.6
Perfluorodecanoic acid (PFDA)	0.2
8:2 Fluorotelomer sulfonate (8:2 FTSA)	0.2
9-chloro-hexadecafluoro-3-oxanonane sulfonate (9Cl-PF3ONS)	0.1
Perfluorononane sulfonate (PFNS)	0.1
Perfluoroundecanoic acid (PFUnDA)	0.5
N-methyl-perfluorooctane sulfonamido acetic acid (MeFOSAA)	0.1
N-ethyl-perfluorooctane sulfonamido acetic acid (EtFOSAA)	0.1
Perfluorodecane sulfonate (PFDS)	0.1
Perfluorododecanoic acid (PFDoDA)	0.3
11-chloro-eicosafluoro-3-oxaundecane-1-sulfonic acid (11Cl-PF3OUdS)	0.1
Perfluorotridecanoic acid (PFTriDA)	0.1
Perfluorotetradecanoic acid (PFTeDA)	1.9

4. ANALYTICAL METHOD

All laboratory glassware used in the extractions was burned at 400 °C overnight and all equipment was rinsed three times with methanol before use. Samples were filtered through glass microfiber filters (47 mm diameter, Whatman™, China), split into two when the analysis was done in duplicate, weighed and spiked with 100 µL of a 50 ng mL^{-1} internal standard mixture in methanol (Wellington Laboratories, MPFAC-24 ES with 13C3-HFPO-DA added individually). The samples were then extracted on Oasis WAX cartridges (6 mL, 150 mg, 30 µm, Waters) as described in Smith *et al.*, 2022[3] and concentrated to 1 mL under nitrogen. Target analysis was done on a SCIEX Triple Quad™ UPLC-MS/MS system (USA) and included 29 target compounds, which are listed in Table S4 together with quality control data. A Phenomenex Kinetix® 1.7 µm C18 precolumn was used to trap PFAS contamination from the mobile phases and LC system prior to extract injection. The organic mobile phase was methanol and the inorganic mobile phase was 10 mM ammonium acetate in Milli-Q water. Extracts were injected on a Phenomenex Gemini® 3 µm C18 HPLC column with a Phenomenex KJ0-4282 analytical guard column, all at 40 °C. MS/MS operation was done in scheduled multiple reaction monitoring (MRM) mode with negative electrospray ionization. Compounds with branched and linear isomers were reported as their summed concentrations. The limit of quantification in the water samples was defined as the lowest concentration with a consistent signal to noise ratio of 10, which was 0.2 ng L^{-1} in the FF samples and 0.4 ng L^{-1} in the EO samples. Of all water samples analyzed for target PFAS in this study (n = 248), the maximum contribution of non-detects to the ΣPFAS concentration was 1.6 %, which was deemed

negligible. Hence, concentrations of PFAS below the LOQ were set to 0. For further details on the analytical method, see Smith *et al*., 2022.[3]

The mean detected masses of each compound over all analyzed blanks (n = 19) are given in Table S4, with values below the LOQ set to zero. This includes laboratory blanks (n = 6), Milli-Q blanks (n = 11) and TOPA blanks (n = 2). For laboratory blanks, IS was spiked directly on a preconditioned cartridge prior to elution. For Milli-Q blanks, 125 mL Milli-Q water was extracted instead of sample. TOPA blanks were 125 mL Milli-Q water on which a TOP assay was performed. Each extraction batch contained at least one blank. In all cases, detected blank concentrations were negligible compared to sample concentrations. For two samples, outlying PFBA concentrations of >730 ng L^{-1} were removed from the analysis, i.e. *n = 3* instead of *4*. Mean concentrations of these samples were 220 ng L^{-1} (min – max: 190 – 260 ng L^{-1}) and 200 ng L^{-1} (min – max: 190 – 230 ng L^{-1}) after removal of these outliers.

Table S4 also gives the mean, min and max recovery of native-spiked Milli-Q and matrix samples (n = 6 for both). Spiked amounts ranged from 5 to 25 ng and matrices included groundwater, leachate, groundwater foam, leachate foam and groundwater electrochemical effluent. For recovery determination, matrix samples were filtered, split in three and one of three samples was spiked with the appropriate amount of a 250 ng mL^{-1} native PFAS mixture in methanol. The remaining two samples were analyzed normally, and the recovery was calculated as the weight-normalized difference in concentration between the spiked extract and the mean of the reference extracts. Most outlying recoveries originated from when low amounts of PFAS were spiked to matrices with high natural PFAS contamination. E.g., when 10 ng PFAS was spiked to a natural extract concentration of 100 ng mL^{-1}, a method variability of 10 % could already cause a recovery variation up to 200 %.

Table S4. Analytical method - quality control data

Compound	*Mean blank detection* $(ng\ mL^{-1}_{Extract})$	*Recovery* $(\%)$, *as mean* $(min - max)$
PFBA	0.20	131 (102-293)
PFPeA	0.25	113 (95-170)
PFBS	0.16	110 (68-154)
PFHxA	0.06	130 (91-231)
4:2 FTSA	<LOQ	98 (59-151)
HFPO-DA	0.01	142 (61-342)
PFPeS	<LOQ	108 (57-275)
PFHpA	0.14	111 (79-178)
NaDONA	<LOQ	141 (76-298)
PFHxS	0.02	103 (63-164)
PFOA	0.05	119 (60-262)
6:2 FTSA	0.59	84 (15-136)
PFHpS	0.01	104 (66-126)
PFECHS	<LOQ	109 (60-170)
PFNA	<LOQ	109 (75-154)
FOSA	0.01	101 (68-167)
PFOS	0.53	90 (44-124)
PFDA	<LOQ	109 (60-185)
8:2 FTSA	0.02	105 (66-150)
9Cl-PF3ONS	<LOQ	74 (25-124)
PFNS	<LOQ	107 (72-187)
PFUnDA	0.02	89 (44-173)
Me-FOSAA	<LOQ	103 (72-136)
Et-FOSAA	<LOQ	97 (64-134)
PFDS	<LOQ	83 (60-108)
PFDoDA	<LOQ	101 (70-154)
11Cl-PF3OUdS	<LOQ	69 (35-102)
PFTriDA	<LOQ	127 (89-186)
PFTeDA	0.01	102 (68-138)

5. TOTAL OXIDIZABLE PRECURSOR (TOP) ASSAYS

A. Methods

After filtration as described in section 4, 2 g potassium persulfate ($K_2S_2O_8$, Sigma Aldrich, USA) and 1.9 mL 10 M sodium hydroxide (NaOH, Sigma Aldrich, USA) were added to each 125 mL sample. For the influent samples of the electrochemical runs with fractionated foam, 50 mL collapsed foam sample was used instead and the amounts of chemicals added were reduced accordingly. Samples were left in a water bath at 85 °C for six hours, cooled in an ice bath and adjusted to a pH of 6-8 by gradually adding 30 % hydrogen chloride (HCl, Merck, Germany). Solid phase extraction and UPLC-MS/MS analysis were subsequently done as for the normal target PFAS analysis.

B. Results

Table S5 shows the mean recoveries of individual PFAA and total PFAS after total oxidizable precursor (TOP) assays for all analyzed samples ($n = 32$). Concentrations prior to oxidation below the LOQ were set to the LOQ, and n indicates the number of samples for which the concentrations in both the reference samples and the oxidized samples were above the LOQ. It should be noted that this choice is somewhat arbitrary and results in a recovery of zero when both the reference and the oxidized sample concentrations are below the LOQ. Since the total concentration of all precursors included in the target analysis was negligible (< 5 % of ΣPFAS in all reference and oxidized samples) and

individual precursor concentrations were often below the LOQ, oxidiation efficiencies were difficult to determine.

The samples included influent and effluent from all FF and EO experiments for both groundwater and leachate, including the foam ($n = 2$ for each sample type). There were no differences in TOP assay recovery between the groundwater and leachate samples, so all sample types were grouped together. Overall, a minor decrease in PFAS concentrations after the TOP assay was measured, which indicates that no considerable concentrations of precursors were present in the groundwater or leachate. Probably, this is because all PFAS originate from the landfill, where any precursors were already degraded due to the high biological activity and long storage time before leaching. The observed decreases in concentrations were small and can be explained by analytical uncertainties.

Table S5. Recovery in TOP assay samples and corresponding standard deviation. When the concentrations in the reference sample prior to oxidation were below LOQ, these were set to the LOQ, such that the denominator was non-zero. n indicates the number of samples for which all concentrations (i.e. before and after oxidation) were above the LOQ.

Compound	*Mean recovery* $\pm$ *sd*	n
PFBA	0.80 ± 0.19	32
PFPeA	0.99 ± 0.19	32
PFHxA	0.99 ± 0.31	32
PFHpA	0.97 ± 0.31	32
PFOA	0.84 ± 0.15	32
PFNA	0.77 ± 0.34	28
PFDA	3.57 ± 8.20	26
PFUnDA	1.07 ± 1.80	17
PFDoDA	0.01 ± 0.07	1
PFTriDA	0.05 ± 0.21	2
PFTeDA	0.09 ± 0.39	2
PFBS	0.93 ± 0.10	32
PFPeS	0.93 ± 0.10	32
PFHxS	0.88 ± 0.07	32
PFHpS	0.80 ± 0.16	32
PFOS	0.87 ± 0.20	32
PFNS	0.21 ± 0.49	6
PFDS	0.01 ± 0.07	1
4:2 FTSA	0.42 ± 0.59	12
6:2 FTSA	0.55 ± 0.51	19
8:2 FTSA	0.45 ± 0.78	11
HFPO-DA	0.94 ± 2.12	8
NaDONA	0.21 ± 0.53	5
PFECHS	0.79 ± 0.12	32
FOSA	1.31 ± 0.91	29
9Cl-PF3ONS	0.01 ± 0.07	1
11Cl-PF3OUdS	0.01 ± 0.07	1
Me-FOSAA	0.51 ± 0.60	18
Et-FOSAA	0.80 ± 0.46	28
ΣPFAS	0.87 ± 0.10	32

6. EXTRACTABLE ORGANOFLUORINE (EOF)

A. Method

In addition to the influent and effluent water samples, three high volume negative blanks (750 mL MilliQ water), three low volume negative blanks (5 mL MilliQ water), three fluoride blanks (750 mL 0.69 mg L^{-1} F^{-}) and five positive blanks (three 750 mL MilliQ water spiked with 50 ng and two 750 mL spiked with 150 ng of each of the 29 PFAS included in the target analysis) were included in the extraction and subsequent analysis. The extraction protocol was the same as described above for the target PFAS analysis, but to account for the higher volume, 500 mg WAX cartridges (6 mL, 500 mg, 60 µm, Waters) were used instead of 150 mg and the second elution step was done with 8 mL instead of 4 mL 0.1 % ammonium hydroxide in methanol.

To minimize background contamination, the sample extract carriers (ceramic boats), containing glass wool for better dispersion of the extracts' fluids, were baked at 1100 °C prior to analysis. Each run started and finished with an 8 points calibration curve (100 µL of 0.025 ng F μL^{-1} to 10 ng μL^{-1} of F^{-} solution, resulting in 2.5 to 1000 ng F^{-} total combusted) and a mid-level calibration standard was run repeatedly to monitor instrumental drift. The calibration curve showed very good linearity with $R^2 > 0.99$. To evaluate combustion efficiency, a mixture of PFOA and PFOS was combusted at the beginning, middle and end of each run. The PFOA and PFOS mixture average recovery was 92 % (n = 6; min – max: 79 % – 104 %), ensuring a good combustion efficiency of the instrument. Aliquots of 50 µL of extracts were combusted.

Combustion of samples was achieved at 1100 °C in a combustion furnace (HF-210, Mitsubishi) under oxygen (400 mL min^{-1}), argon (200 mL min^{-1}) and argon mixed with water vapour (100 mL min^{-1}) flow for around 5 min. Combustion gases were absorbed in MilliQ water using a gas absorber unit (GA-210, Mitsubishi), 200 µL aliquots of the absorption solution were injected onto the ion chromatograph (Dionex Integrion HPIC, Thermo Fisher Scientific) equipped with an anion exchange column (Dionex IonPac™ AG19-4µm 2x50 mm guard column and Dionex IonPac™ AS19-4µm 2x250 mm analytical column) operated at 35 °C. Chromatographic separation was achieved by running a gradient of aqueous hydroxide mobile phase ramping from 8 mM to 60 mM at a flow rate of 0.25 mL min^{-1} and fluoride was detected by a conductivity detector.

No differences between the low volume and high volume extraction blanks were observed, with an average of 9 ng F combusted, extrapolated to 49 ng L^{-1} in the water. Therefore, the mean EOF concentration in the extraction blanks (n = 6, 3 x 5 mL and 3 x 750 mL) was subtracted from the samples in all cases. The LOQ for EOF analysis was defined as the average concentration plus three times the standard deviation in the method blanks (62.6 ng F L^{-1}) and no samples were found to be below it. The known EOF concentrations based on the measured target PFAS concentrations were calculated as described in Schultes *et al*. (2018).[4]

B. Results and quality control

The mean recovery of positive blanks (50 ng or 150 ng of the 29 PFAS included in the targeted analysis) was 70 % (n=5; min – max: 62 % – 92 %), ensuring satisfactory EOF recovery. Recovery of fluoride in the fluoride blanks was only 0.45 % (n = 3; min – max: 0.18 % – 0.59 %). Nonetheless, since the fluoride concentration in the water samples ranged between <0.1 mg L^{-1} and 0.69 mg L^{-1} (Tables S1 and S2), fluoride may have contributed to the measured EOF concentrations, which were between 0.2 and 14 µg L^{-1}. When assuming the mean fluoride recovery of 0.45 %, the theoretical contribution of fluoride to the measured EOF ranged between 19 % and 1261 % (mean: 192 %). However, no significant correlation between aqueous fluoride concentrations and EOF concentrations were found (p = 0.23, fluoride concentrations below LOQ were set to the LOQ). This lack of correlation indicates that the fluoride removal during sample extraction was sufficient, but the exact effect of fluoride on the EOF concentrations remains uncertain.

A quantitative comparison between the measured EOF concentrations and the known EOF from the target PFAS concentrations was difficult to make. In the target PFAS analysis, concentrations were determined based on the peak area ratio with corresponding internal standards (IS). Due to the nature of the method, EOF concentrations were not IS-corrected, which means that target PFAS concentrations were not directly comparable to EOF concentrations. If EOF concentrations were corrected using the mean recovery of the spiked blanks, EOF concentrations were on average 82 % higher (min – max: 11 % – 358 %) than the known EOF concentration from the target PFAS concentrations. If EOF concentrations were not recovery-corrected, the concentrations were on average 27 % higher (min – max: -23 % – 220 %) than expected. Since the combustion efficiency and EOF recovery varied, the real difference is probably somewhere in between these values. Over all samples, a significant correlation between the known EOF from the target PFAS and the measured EOF was found (Pearson's r = 0.89, $p \approx 10^{-11}$), indicating that target PFAS concentrations were good indicators of the relative EOF concentrations.

The higher-than-expected EOF concentrations can be explained by the presence of extractable PFAS that were not included in our targeted method, and that do not degrade to target PFAS in the TOP assay. Examples of such are ultrashort-chain,[5] unsaturated[6] or chlorinated PFAS.[7] Fluorinated organic molecules that are not perfluorinated may have contributed to the measured EOF concentrations as well. The contribution of ultrashort-chain PFAS to the EOF content is uncertain. Generally, extra washing steps are included in the extraction procedure for EOF to remove fluoride, with the drawback that ultrashort-chain PFAS are lost as well.[8] Since we used the same extracts for bioassay and EOF analysis, these extra washing steps were omitted (but fluoride recovery was nonetheless only 0.45 %, as outlined above). Hence, more short-chain PFAS may have been recovered in the extracts and contributed to the measured EOF concentrations than is generally the case in EOF analysis. Efforts to identify these unknown fluorinated compounds with non-target methods are currently ongoing.

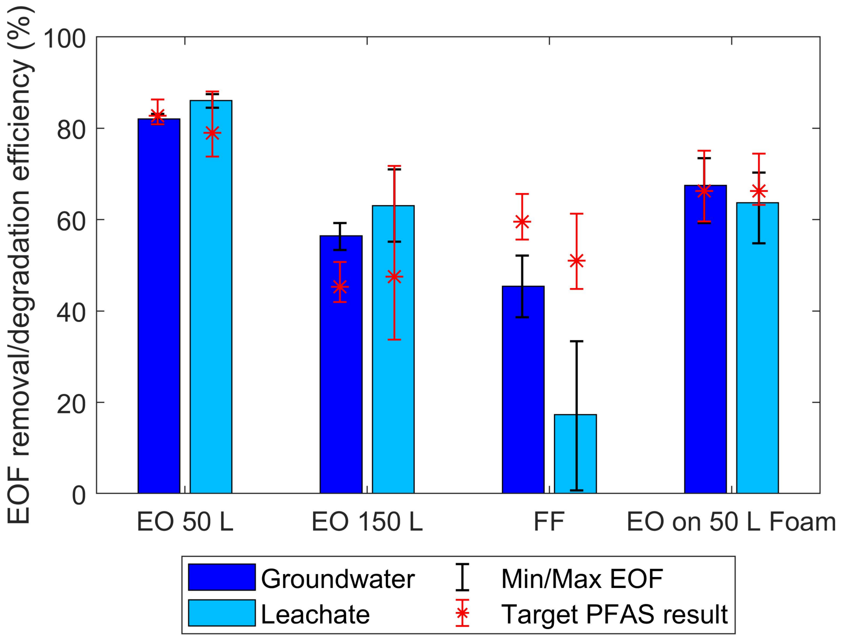

Figure S3. EOF removal (FF) or degradation (EO) efficiency of the different treatments, compared to the result based on total target PFAS concentrations. Black error bars represent the min/max of the EOF results, red error bars the min/max of the target PFAS results, and red asterisks the mean target PFAS result.

The EOF degradation efficiencies in the EO tests with 50 L volume were similar to the degradation of target PFAS, see Figure S3. Conversely, the mean EOF degradation was slightly higher than the target PFAS degradation at 150 L volume. Possibly, this indicates the formation of fluorinated byproducts in the EO at a high specific charge, i.e. at lower treated volumes. Accordingly, the EOF degradation would be higher than the target PFAS degradation at all treatment volumes, due to the presence of more degradable substances such as unsaturated or chlorinated PFAS. However, in the 50 L tests, this higher PFAS degradation is cancelled out by the formation of fluorinated byproducts. This hypothesis is strengthened by the slightly lower toxicity measured in the TTR-binding assay on the effluent of the 150 L EO experiments as compared to the 50 L experiments, see main text Figure 4. However, measurement uncertainties were high and experiments were only done in duplicate, so more research is needed to look further into the formation of fluorinated byproducts.

Conversely, the EOF removal in the foam fractionation was on average 45 % lower than the removal of target PFAS. This indicates the presence of PFAS that are not susceptible to removal by FF, such as ultrashort-chain PFAS or non-amphiphilic compounds.

7. BIOASSAYS

In addition to the influent and effluent water extracts, the high volume negative blanks and the high concentration positive blanks (150 ng of each PFAS included in the target analysis in 750 mL MilliQ water) were included in both bioassays as well.

A. Method - TTR binding assay

Transthyretin-binding experiments were performed in black 96-well polystyrene nonbinding plates (Greiner Bio-One) as described in Hamers *et al.*, 2020.[9] Briefly, a fluorescent conjugate of T_4 and fluorescein 5-isothiocyanate (FITC) was used. This FITC-T_4 conjugate emits increased fluorescence when bound to TTR, so the presence of TTR-binding compounds in an incubated extract will lead to a decreased fluorescence emission compared to a blank incubation. The fluorescence was measured twice for each extract in duplicate sample dilution curves and the data were fit to dose-response curves, to derive the concentration of sample extract (% of well volume) causing 50 % inhibition of TTR-binding by FITC-T_4 (EC_{50}). Using the EC_{50} value of PFOS (289 nM), PFOS-equivalent concentrations in the original water samples were calculated, i.e. a theoretical aqueous concentration of PFOS that in the same dilution as the extract would lead to the EC_{50} determined for the extract.

Duplicate sample dilution curves were prepared at 1, 0.3, 0.1, 0.03, 0.01, 0.003, 0.001 and 0 % of the final total well volume (200 µL). The methanol fraction was kept constant at 1 % of the total well volume for all dilutions. Initial dilutions in 100 µL Tris-HCl buffer at pH 8 were shaken for 5 min at 900 rpm and the background fluorescence was measured in arbitrary fluorescence units (AFU) at λ_{ex} = 490 nm and λ_{em} = 518 nm. Subsequently, 50 µL 110 nM fluorescein 5-isothiocyanate thyroxine (FITC-T_4) in Tris-HCl buffer was added to each well and the fluorescence was measured again after shaking for 5 min. Finally, 50 µL 0.12 µM transthyretin (TTR) in TRIS-HCl buffer was added and the fluorescence was measured in the presence of both FITC-T_4 and TTR. Influent and effluent samples from

each experiment were always incubated on the same 96-well plate.

The fraction of FITC-T_4 bound to TTR (f_{bound}) at each dilution i was calculated according to Equation S3, with F_{TTR} and F_{FT4} the fluorescence after addition of TTR and FITC-T_4, respectively. These data were fit to the inhibition curve given in Equation S4, with x the sample concentration (% of well volume), by minimizing the sum of squared errors between the calculated data $f_{bound,calc}$ and average measured data f_{bound} to find optimized values for the EC_{50} (% of well volume) and hill slope s.

$$f_{bound,i} = \frac{[F_{TTR} - F_{FT4}]_{sample\ i}}{[F_{TTR} - F_{FT4}]_{blank}} \cdot 100\% \tag{S3}$$

$$f_{bound,calc} = \frac{100}{1 + (\frac{x}{EC_{50}})^s} \tag{S4}$$

The measured EC_{50} values (% well volume) were subsequently expressed in PFOS-equivalent concentrations in the original sample as per Equation S5, with $EC_{50,PFOS}$ the average optimized EC_{50} value of PFOS ($2.89 \cdot 10^{-7}$ M). The factor 3750 is the concentration factor from sample to extract (0.75 L to 200 µL). The expected PFOS-equivalent concentration of each sample was calculated as in Equation S6, with C_i and $EC_{50,ref,i}$ the original aqueous concentration (M) and predetermined EC_{50} (M) of compound i, respectively. For compounds without a predetermined EC_{50} value (*n = 10*, with a total contribution to the mean influent $\Sigma PFAS$ concentration of 10 %) the EC_{50} value of PFDS was assumed, which was the highest value of all included compounds (34.8 µM). Because each experiment was carried out in duplicate and each extract was analyzed in duplicate, n = 4 for each data point, but these data points are not independent. Hence, statistical significance was not calculated, because the independent sample size was only 2.

$$C_{PFOS-eqv,\ meas}(M) = \frac{100}{3750} \cdot \frac{EC_{50,PFOS}}{EC_{50,extract}} \tag{S5}$$

$$C_{PFOS-eqv,\ calc}(M) = \sum \frac{C_i \cdot EC_{50,PFOS}}{EC_{50,ref,i}} \tag{S6}$$

B. Method - *A. fischeri* bioluminescence assay

White lumitrac polystyrene 96-well plates (Greiner Bio-One) were washed under hot tap water and rinsed thoroughly with demineralized water. Duplicate dilution curves at 1, 0.33, 0.1, 0.033, 0.01 and 0.033 % of the total well volume (200 µL) were prepared in 100 µL 50 mM KPi buffer with 2 % NaCl. The methanol fraction was kept constant at 1 %. 100 µL *A. fischeri* bacteria suspension in NaCl buffer, prepared according to the instructions from the provider (Modern Water), was automatically injected into each well, and the luminescence was measured after 15 and 30 minutes. These results were similar, so only the results after 30 min exposure are shown. Each plate also contained duplicate dilution curves containing 200, 60, 20, 6, 2, 0.6, 0.2 and 0.06 µM triclosan in 1 % DMSO in NaCl buffer, as well as duplicate DMSO and MeOH blanks. The differences in luminescence between the samples and the blanks were fitted to Equation S4 and expressed in triclosan equivalent concentrations identically as for the TTR-binding tests, using the triclosan EC_{50} measured on the corresponding plate.The antibacterial agent triclosan was used for the expression of the toxicity because an EC_{50} of PFOS individually could not be determined with the *A. fischeri* assay.

C. Quality control - TTR binding assay

All extracts from the untreated groundwater and leachate had a yellow color, leading to a visible coloring of the wells with high extract concentration. The blank fluorescence measurements, i.e. before the addition of FITC-T_4, showed non-zero values in the wells with high sample concentrations. However, there was no significant difference between the increase in fluorescence after FITC-T_4 addition to samples with and without background fluorescence, indicating that the total fluorescence is additive in nature. The mean EC_{50} values of the negative and positive control extracts as well as of pure methanol and a 29 µg L^{-1} ΣPFAS stock solution in methanol were all at least 4 times higher than that of the least active effluent sample when all were expressed in PFOS-eq. Negative controls often yielded linear response curves, which occurred when the highest concentration (% well volume) did not generate a measurable response, in which case EC_{50} values could not be determined. The low response of the positive controls confirmed that PFAS were not the primary cause of the TTR-binding activity of the water samples.

D. Quality control - *A. fischeri* bioluminescence assay

To assess any decrease in luminescence caused by light absorption rather than mortality of the bacteria, the absorption of each extract dilution curve at 490 nm wavelength was measured.[10] *A. fischeri* emit light around this wavelength[11] and from the absorption data, the percentage of light that is transmitted through the well was calculated according to Equation S7. All data are given in Table S6 and the transmission was always higher than 85 %, indicating that toxicity is the dominating cause for decreased light emission. However, the electrochemical influent always had a higher absorption than the effluent, which may have contributed to the difference in toxicity measured between electrochemical influent and effluent samples.

$$\%_{transmitted} = 10^{2 - Abs_{Sample} + Abs_{Blank}} \tag{S7}$$

For the *A. fischeri* bioluminescence assay, measurable activity was also found in the positive and negative control extracts, although both at least two times lower than in any of the sample extracts. The negative and positive controls had mean triclosan equivalent concentrations of 7 and 10 nM respectively, compared to 22 nM in the sample with the next lowest activity (groundwater 150 L EO effluent). The measurable activity in the negative (Milli-Q) control indicates that some of the sample response may have come from the extraction procedure. The similar activities in the negative and positive controls indicate that PFAS are not mainly responsible for the response in the *A. fischeri* bioluminescence assay.

Table S6. **Transmission of light at 490 nm (%) through *A. fischeri* bioluminescence sample dilution curves. EO: electrochemical oxidation, FF: foam fractionation, GW: groundwater, Leach: leachate, I: influent, E: effluent**

Sample	1 %	0.33 %	0.10 %	0.03 %	0.01 %	0.003 %
EO GW 50L I1	92.9	98.6	100	102	101	102
EO GW 50L I2	92.5	98.1	100	101	101	102
EO GW 50L E1	100	101	101	102	102	102
EO GW 50L E2	99.9	101	101	102	102	102
EO Leach 50L I1	89.8	96.9	99.7	101	102	101
EO Leach 50L I2	87.4	95.9	98.6	101	102	101
EO Leach 50L E1	101	102	102	102	102	101
EO Leach 50L E2	100	100	100	100	100	100
EO GW 150L I1	93.5	98.6	99.4	102	102	102
EO GW 150L I2	94.3	98.6	99.6	102	102	102
EO GW 150L E1	101	102	102	102	102	102
EO GW 150L E2	101	102	102	103	102	103
EO Leach 150L I1	88.4	96.4	99.6	101	101	101
EO Leach 150L I2	88.2	96.5	99.7	101	99.2	101
EO Leach 150L E1	101	103	102	102	102	101
EO Leach 150L E2	102	102	102	102	102	101
FF GW I1	95.0	98.3	100	101	101	101
FF GW I2	95.5	98.5	100	101	101	101
FF GW E1	95.6	98.8	100	101	101	101
FF GW E2	95.5	97.3	99.6	101	101	101
FF Leach I1	89.8	95.4	99.2	100	101	101
FF Leach I2	89.7	96.8	99.0	100	98.4	101
FF Leach E1	90.0	96.7	98.8	101	101	101
FF Leach E2	91.1	97.5	98.8	101	101	101
EO GW Foam I1	96.8	97.7	100	101	101	100
EO GW Foam I2	95.5	98.5	100	100	101	101
EO GW Foam E1	101	101	101	101	101	101
EO GW Foam E2	101	101	101	100	100	101
EO Leach Foam I1	90.8	97.0	99.2	100	100	101
EO Leach Foam I2	91.3	97.4	99.0	100	100	101
EO Leach Foam E1	101	101	101	101	101	101
EO Leach Foam E2	101	101	100	101	101	101
Mean of blanks	99.2	99.6	99.4	99.9	100	99.8
Methanol blanks – not diluted			100	99.4	100	100

8. MODEL EQUATIONS

The coupled numerical model to describe the degradation of PFAS and the formation of degradation products (that are subsequently degraded) is based on the design of the electrochemical cell (Figure S4). The model will be described by first deriving the ordinary differential equations (ODEs) to describe concentration change in the electrochemical cell (section A), discretizing the ODEs (section B), and finally coupling the parent compounds to the transformation compounds (Section C)

A. From mass balance to ODEs

First, the concentration in the inlet tank (C_1) is calculated from a simple mass balance based on inflow and outflow. It is assumed that there is no reactive degradation of PFAS here. The concentration in the inflow into the inlet tank is equal to the concentration in the effluent from the electrochemical cell, which is denoted as C_N. The mass balance over the inlet tank can then be written as follows, with V_1 the volume of the inlet tank (L) and Q the flow rate $(L\,min^{-1})$:

$$V_1 \cdot \frac{dC_1}{dt} = Q \cdot C_N - Q \cdot C_1 \tag{S8}$$

$$\frac{dC_1}{dt} = \frac{Q}{V_1} \cdot C_N - \frac{Q}{V_1} \cdot C_1 \tag{S9}$$

The concentration in the electrochemical cell is a function of both time and place in the reactor, hence the ordinary differential equations are derived from a flux balance. A denotes the cross-sectional area of the reactor and v the velocity of the flow, both are assumed constant in time as well as space. R is the reaction term, which is also a function of time and space. A small segment of the electrochemical cell between z and $z + dz$ is considered:

$$A \cdot dz \cdot \frac{dC(t,z)}{dt} = v_z \cdot A \cdot C_{z,t} - v_{z+dz} \cdot A \cdot C_{z+dz,t} + R \cdot d_z \cdot A \tag{S10}$$

$$\frac{dC(t,z)}{dt} = \frac{v_z \cdot A \cdot C_{z,t} - v_{z+dz} \cdot A \cdot C_{z+dz,t}}{A \cdot dz} + R \tag{S11}$$

Implementing the assumption that v is constant over space as well as time, this can be rewritten as:

$$\frac{dC(t,z)}{dt} = -v \cdot \frac{dC(t,z)}{dz} + R \tag{S12}$$

B. Discretization of ODEs

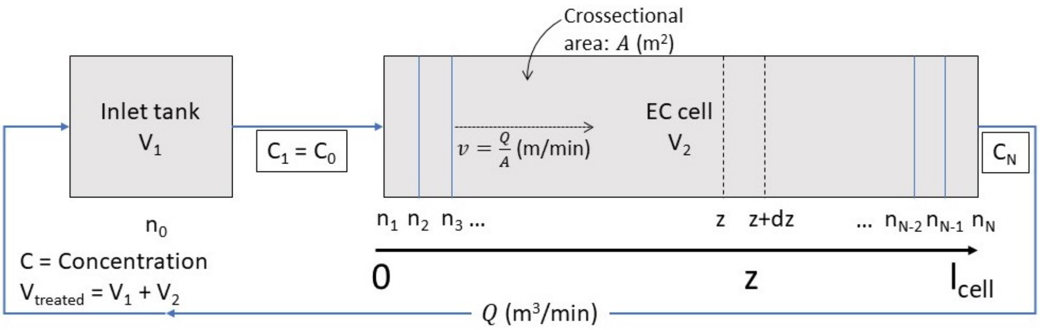

Figure S4. Discretization of the electrochemical reactor

The electrochemical cell is divided into N different nodes over its length, denoted by the subscript n, as illustrated in Figure S4. The concentration in node 1 is assumed equal to the concentration in the inlet tank. The differential equation for the concentration in node 1 is thus equal to Equation S9. The ODE for the concentration in the remaining nodes is:

$$\frac{dC_n}{dt} = \frac{Q}{A_{cell}} \cdot [\frac{dC}{dz}]_n + R_{n-1} \tag{S13}$$

$\frac{dC}{dz}$ for nodes $n = 2 \ldots N$ is calculated using a backward difference as:

$$[\frac{dC}{dz}]_n = \frac{C_n - C_{n-1}}{z_n - z_{n-1}} \tag{S14}$$

and implemented in the equation above.

C. Coupling of ODEs for different compounds

The reaction term R is different for each compound and consists of the sum of formation and degradation reactions. Electrochemical PFAS degradation follows a step-wise pathway, where the chain is shortened by subsequent loss of one CF_2 group.[12] As explained in the main text, perfluorosulfonic acids (PFSA) can degrade to shorter chain PFSA as well as to perfluorocarboxylic acids (PFCA). Conversely, PFCA only degrade to shorter chain PFCA. Finally, formation of both PFCA and PFSA from precursors can occur. Because the TOP assay results did not indicate the presence of any PFAA precursors, PFOS and PFOA precursor concentrations and kinetic constants were set to zero, and no other PFAA precursors were included in the model. For all PFSA, the reaction term then equates to

$$R_i(n) = -k_i \cdot C_i(n-1) + k_{i+1} \cdot C_{i+1}(n-1) \tag{S15}$$

with C_i and k_i the concentration of the PFSA with chain length i and the kinetic constant of the degradation of that same PFSA, respectively. Similarly, C_{i+1} and k_{i+1} are the concentration and kinetic constant for degradation of the PFSA with chain length $i+1$, respectively. For PFCA, additionally, degradation reactions of PFSA with the same chain length are added as formation reactions to this term, each with a corresponding kinetic constant.

As an example, for PFHpA, the total discretized ODE in nodes $n = 2 \ldots N$ is then given as:

$$\begin{aligned} \frac{dC_{PFHpA}}{dt}(z,t) = Q/A_{cell} \cdot \frac{C_{PFHpA,n} - C_{PFHpA,n-1}}{z_n - z_{n-1}} \ldots \\ - k_{PFHpA} \cdot C_{PFHpA,n-1} + k_{PFOA} \cdot C_{PFOA,n-1} + k_{PFHpS \rightarrow PFHpA} \cdot C_{PFHpS,n-1} \end{aligned} \tag{S16}$$

An overview of all kinetic constants included in the model is given in Table S7. As described in the methods section of the main text, the constants were obtained by minimization of the sum of squared errors between the model and experimental results. These kinetic constants are observational and may represent multiple combined reactions, each following a slightly different mechanism but leading to the same degradation products.[12] All reactions given in this table were included in the model, but some of the rate constants were set to zero to exclude certain degradation pathways. Formed perfluoropropanoic acid (PFPrA) concentrations were used for checking of the mole balance, which closed for every simulation. The entire model code is available on request to the corresponding author.

Table S7. Kinetic constants included in the model. The constants calibrated based on the results from the run with 50 L groundwater were used in all model simulations, but separate constants were also calibrated for the experiments with fractionated foam. Certain reaction pathways were excluded by setting the corresponding kinetic constant to 0. It should be noted that these rate constants depend on the current and will be different at current intensities other than the one used in this study (231 A, 25 mA cm^{-2}).

k	*Reaction*	*Value* (min^{-1})	*Value* (min^{-1})	*Value* (min^{-1})
	Calibration using	*GW 50 L*	*Leach Foam*	*GW Foam*
k_1	*Precursors → PFOA*	0	0	0
k_2	*Precursors → PFOS*	0	0	0
k_3	*PFOS → PFHpS*	0	0	0
k_4	*PFHpS → PFHxS*	0	0	0
k_5	*PFHxS → PFPeS*	0.0032	0.0009	0.0001
k_6	*PFPeS → PFBS*	0.0105	0.0019	0.0000
k_7	*PFBS → PFPrS*	0	0	0
k_8	*PFOS → PFOA*	0.0092	0.0000	0.0009
k_9	*PFHpS → PFHpA*	0.0059	0.0000	0.0011
k_{10}	*PFHxS → PFHxA*	0	0	0
k_{11}	*PFPeS → PFPeA*	0	0	0
k_{12}	*PFBS → PFBA*	0.0047	0.0022	0.0009
k_{13}	*PFOA → PFHpA*	0.0169	0.0105	0.0101
k_{14}	*PFHpA → PFHxA*	0.0402	0.0276	0.0297
k_{15}	*PFHxA → PFPeA*	0.0341	0.0350	0.0384
k_{16}	*PFPeA → PFBA*	0.0560	0.0647	0.0694
k_{17}	*PFBA → PFPrA*	0.0484	0.0340	0.0481

D. Pseudocode

The ODEs describing the degradation or formation of each compound are combined in the pseudocode shown in Algorithm S1. This set of ODEs is solved in Matlab R2020b using the ode23 solver. Its output is a matrix containing the stacked concentrations of all compounds over time (rows) and axial position (columns). The size of this matrix is $n_{time} \times (n_{compounds} \cdot N)$, with n_{time} the number of time points evaluated, $n_{compounds}$ the number of compounds included in the model and N the length of the position vector. Restructuring this matrix appropriately yields the $n_{compounds} \times n_{time}$ matrix containing the concentrations of all compounds in the inlet tank over time.

Algorithm S1. Model Pseudocode

```
procedure MYODECOUPLED(Q, V1, Acell, k, z, C)
    dCdt(1) = Q/V1 · (C(end) − C(1))                          ▷ set dC/dt in first node (inlet tank)
    for i ∈ 1 : 13 do                                          ▷ Loop over the number of included compounds
        for n ∈ 2 : N do                                       ▷ Loop over the length of the z vector
            dCdz(i, n) = (C(n) − C(n − 1))/(z(n) − z(n − 1))
            dCdt(i, n) = −Q/Acell · dCdz(i, n) + R(i, n)
                                                               ▷ R defines the reaction term for each compound i at node n
    Reshape(dCdt)
                                                               ▷ Save C as column vector that stacks the ODEs for each compound at each node n
```

9. ELECTROCHEMICAL OXIDATION - ADDITIONAL RESULTS

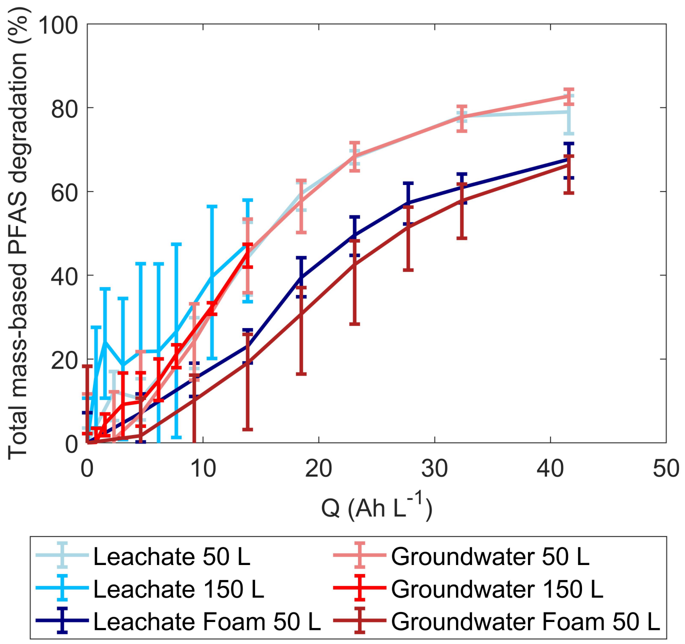

Figure S5. ΣPFAS degradation versus specific charge Q

The specific charge Q (Ah L^{-1}) is defined in equation S17, with V the total treated volume (L), t the treatment time (h) and J the current (A). Q can be used to calculate the energy consumption ($W,\ kWh\,m^{-3}$) of the electrochemical oxidation, as per equation S18, with U (V) the time-averaged voltage of the cell (see figure S1). This can subsequently be normalized per log removal of PFOS or PFOA using equation S19 (W_n), with C_0 and C_{end} the PFOS/PFOA concentration at the start and end of the EO treatment, respectively. For the log-normalization of the energy demand over the entire treatment train, C_0 was set to the concentration in the influent to the FF process, and C_{end} to the concentration in the effluent from the EO on the fractionated foam. The so obtained value for W_n was then multiplied with 0.1, since only 10 % of the influent water ended up as foam and was thus subjected to EO treatment.

$$Q = \frac{t \cdot J}{V} \tag{S17}$$

$$W = Q \cdot U \tag{S18}$$

$$W_n = \frac{W}{log_{10}(C_0/C_{end})} \tag{S19}$$

Table S8. Mean PFAS concentrations in the groundwater before treatment and after each treatment step. The foam concentrations reported here include the samples taken during the FF experiment (n = 4) as well as the samples from the bulk foam prior to EO (n = 4), whereas degradation efficiencies during EO were calculated based on the concentrations of the bulk foam exclusively.

PFAS	*Untreated*	*FF Effluent*	*EO* 50 *L Effluent*	*EO* 150 *L Effluent*	*Foam*	*EO Foam Effluent*
	$(n = 12)$	$(n = 4)$	$(n = 4)$	$(n = 4)$	$(n = 8)$	$(n = 4)$
PFBA	220	240	30	150	200	300
PFPeA	250	290	14	140	330	200
PFBS	170	190	140	130	310	360
PFHxA	500	470	13	210	1400	190
4:2 FTSA	0.45	<LOQ	<LOQ	0.1	0.092	<LOQ
HFPO-DA	<LOQ	<LOQ	<LOQ	<LOQ	1.9	<LOQ
PFPeS	59	28	59	66	410	580
PFHpA	260	78	11	140	1800	230
NaDONA	<LOQ	<LOQ	<LOQ	<LOQ	<LOQ	<LOQ
PFHxS	350	43	160	280	2700	2600
PFOA	930	88	17	230	8300	500
6:2 FTSA	8.4	<LOQ	2.2	3.4	110	<LOQ
PFHpS	19	1.3	4.6	13	160	140
PFECHS	33	3.8	11	22	370	370
PFNA	12	0.66	<LOQ	2	120	6.4
FOSA	1.5	0.3	0.16	<LOQ	10	0.26
PFOS	220	15	29	100	1800	930
PFDA	1.5	1.7	<LOQ	<LOQ	14	0.22
8:2 FTSA	<LOQ	0.81	<LOQ	<LOQ	1	<LOQ
9Cl-PF3ONS	<LOQ	<LOQ	<LOQ	<LOQ	<LOQ	<LOQ
PFNS	<LOQ	<LOQ	<LOQ	<LOQ	0.22	0.79
PFUnDA	0.23	2.5	<LOQ	0.24	0.073	<LOQ
Me-FOSAA	0.8	<LOQ	<LOQ	<LOQ	5.5	<LOQ
Et-FOSAA	4.9	0.46	0.1	0.7	27	0.86
PFDS	<LOQ	<LOQ	<LOQ	<LOQ	<LOQ	<LOQ
PFDoDA	0.17	1.4	0.15	<LOQ	<LOQ	<LOQ
11Cl-PF3OUdS	<LOQ	<LOQ	<LOQ	<LOQ	<LOQ	<LOQ
PFTriDA	<LOQ	0.4	<LOQ	<LOQ	<LOQ	<LOQ
PFTeDA	0.15	0.13	0.43	<LOQ	<LOQ	<LOQ

Table S9. Mean PFAS concentrations in the leachate before treatment and after each treatment step. The foam concentrations reported here include the samples taken during the FF experiment (n = 4) as well as the samples from the bulk foam prior to EO (n = 4), whereas degradation efficiencies during EO were calculated based on the concentrations of the bulk foam exclusively.

PFAS	*Untreated* $(n = 12)$	*FF Effluent* $(n = 4)$	*EO 50 L Effluent* $(n = 4)$	*EO 150 L Effluent* $(n = 4)$	*Foam* $(n = 8)$	*EO Foam Effluent* $(n = 4)$
PFBA	200	180	67	130	150	150
PFPeA	230	200	13	110	190	65
PFBS	120	110	95	110	150	140
PFHxA	390	330	26	160	560	59
4:2 FTSA	0.48	0.53	<LOQ	<LOQ	0.92	<LOQ
HFPO-DA	0.88	<LOQ	<LOQ	<LOQ	<LOQ	<LOQ
PFPeS	22	12	24	28	57	77
PFHpA	210	69	20	120	450	80
NaDONA	0.025	<LOQ	<LOQ	<LOQ	0.1	<LOQ
PFHxS	120	17	77	110	200	230
PFOA	570	54	49	260	780	160
6:2 FTSA	15	2.7	2.9	13	29	4
PFHpS	9.6	0.62	3.9	8.6	10	13
PFECHS	32	3.1	17	30	42	48
PFNA	11	1	1.2	4.7	15	4.2
FOSA	2.6	0.38	0.22	<LOQ	3.1	0.42
PFOS	190	11	41	120	160	150
PFDA	4.4	0.56	0.26	1.1	5	0.62
8:2 FTSA	0.28	0.27	<LOQ	0.14	1.1	<LOQ
9Cl-PF3ONS	<LOQ	<LOQ	<LOQ	<LOQ	<LOQ	<LOQ
PFNS	0.034	<LOQ	<LOQ	<LOQ	0.011	<LOQ
PFUnDA	0.45	0.21	0.65	0.29	0.83	0.11
Me-FOSAA	4.1	0.55	<LOQ	0.38	4.8	<LOQ
Et-FOSAA	18	2.3	0.87	2.6	20	0.55
PFDS	<LOQ	<LOQ	<LOQ	<LOQ	<LOQ	<LOQ
PFDoDA	1.3	0.16	1.9	0.2	0.2	<LOQ
11Cl-PF3OUdS	<LOQ	<LOQ	<LOQ	<LOQ	<LOQ	<LOQ
PFTriDA	0.19	0.11	0.68	<LOQ	<LOQ	<LOQ
PFTeDA	0.75	0.11	2.1	0.11	0.079	0.23

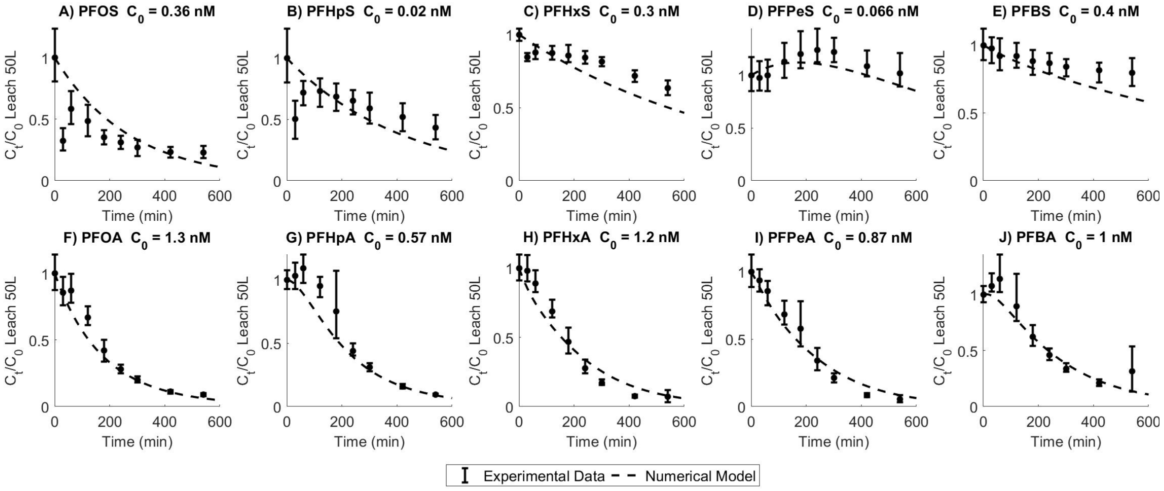

Figure S6. Individual degradation of PFSA and PFCA with chain lengths up to 8 for the EO run with 50 L leachate. Error bars represent min and max values based on the experimental and analytical duplicates (i.e. n = 4), dots represent the means and the dotted line is the model prediction with calibrated kinetic constants, see Table S7.

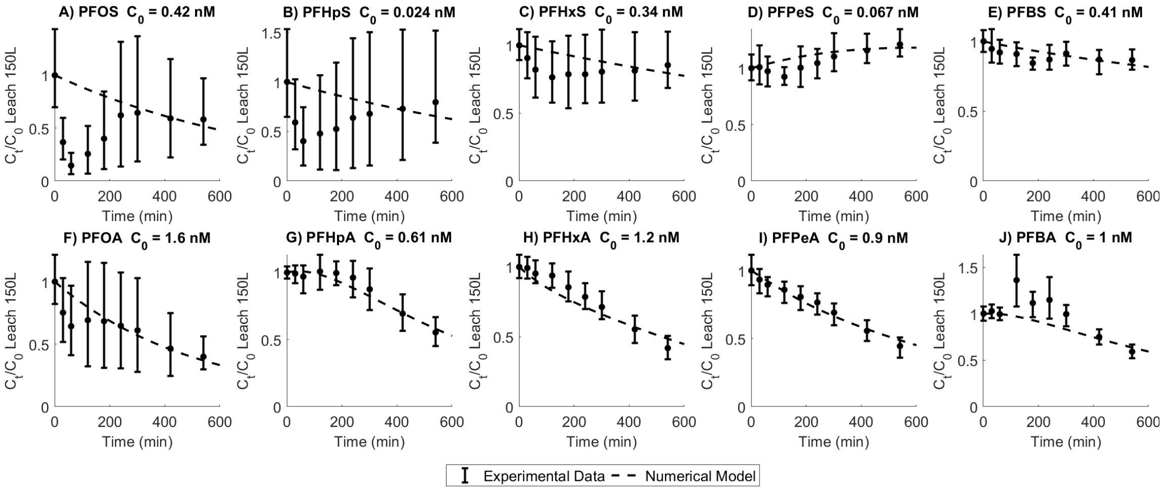

Figure S7. Individual degradation of PFSA and PFCA with chain lengths up to 8 for the EO run with 150 L leachate. Error bars represent min and max values based on the experimental and analytical duplicates (i.e. n = 4), dots represent the means and the dotted line is the model prediction with calibrated kinetic constants, see Table S7.

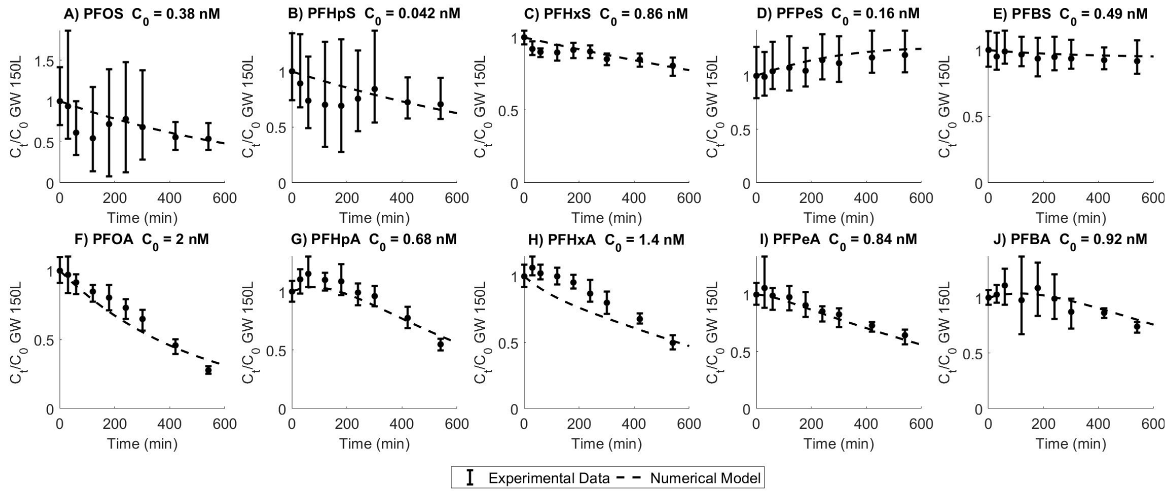

Figure S8. Individual degradation of PFSA and PFCA with chain lengths up to 8 for the EO run with 150 L groundwater. Error bars represent min and max values based on the experimental and analytical duplicates (i.e. n = 4), dots represent the means and the dotted line is the model prediction with calibrated kinetic constants, see Table S7.

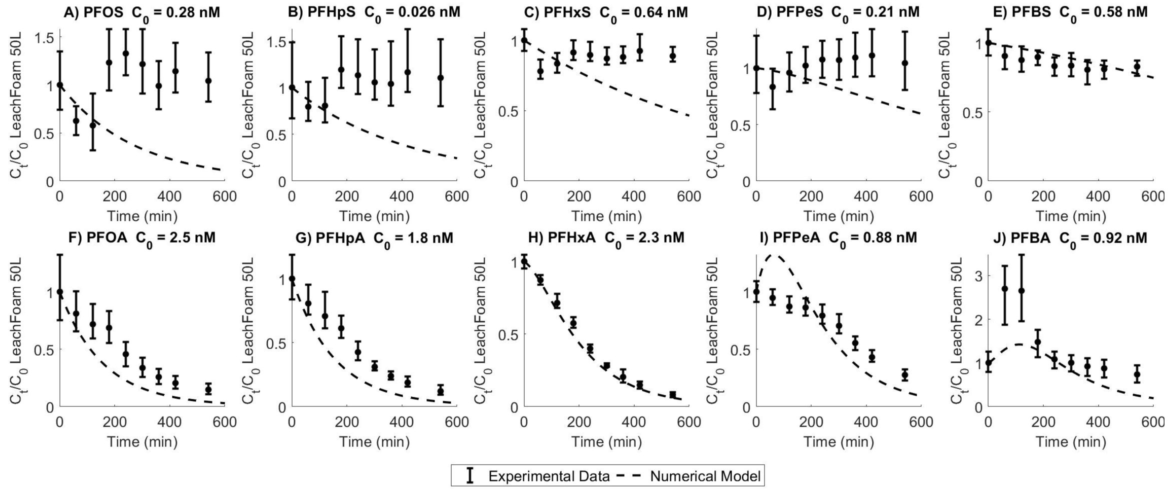

Figure S9. Individual degradation of PFSA and PFCA with chain lengths up to 8 for the EO run with 50 L leachate foam, using the kinetic constants calibrated based on the results from the 50 L GW experiment. Error bars represent min and max values based on the experimental and analytical duplicates (i.e. n = 4), dots represent the means and the dotted line is the model prediction with calibrated kinetic constants, see Table S7.

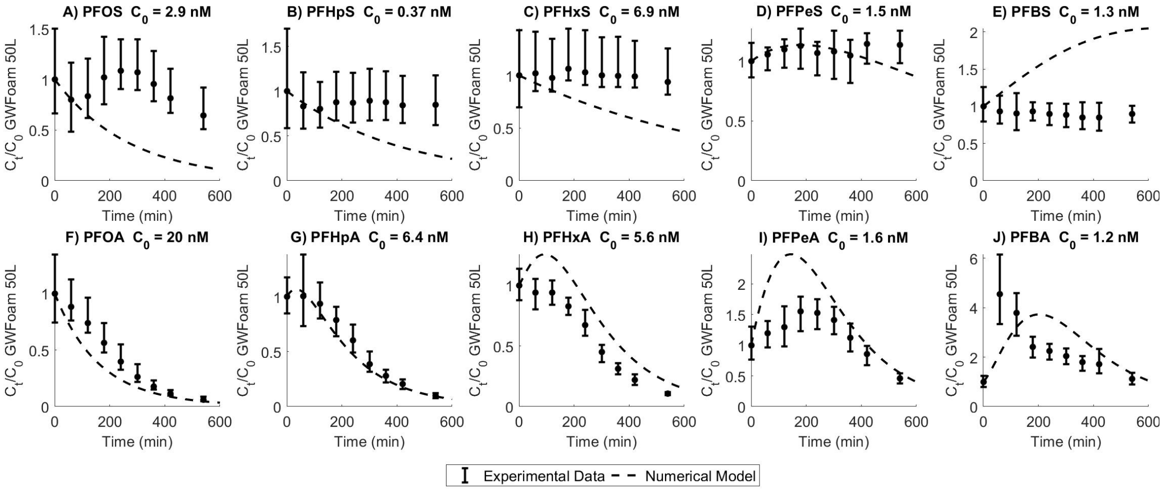

Figure S10. Individual degradation of PFSA and PFCA with chain lengths up to 8 for the EO run with 50 L groundwater foam, using the kinetic constants calibrated based on the results from the 50 L GW experiment. Error bars represent min and max values based on the experimental and analytical duplicates (i.e. n = 4), dots represent the means and the dotted line is the model prediction with calibrated kinetic constants, see Table S7.

Figures S11 and S12 show the model fits when the kinetic constants were calibrated based on the results from each EO experiment with fractionated foam. These kinetic constants are given in the last two columns of Table S7. The degradation rate constants of especially long-chain PFCA and PFSA were much lower than the constants calibrated based on the experiment with 50 L GW. This indicates that the reaction may have been hindered by matrix effects, electrode scaling or a change in pathway. Moreover, the higher initial concentrations may have caused a shift to a reaction-limited degradation, where zero-order kinetics are more appropriate. The concentrations of short-chained PFCA are still not reproduced very accurately, indicating that the degradation pathway may not be included correctly. More fundamental research into the degradation mechanism of PFSA is needed to refine this part of the model.

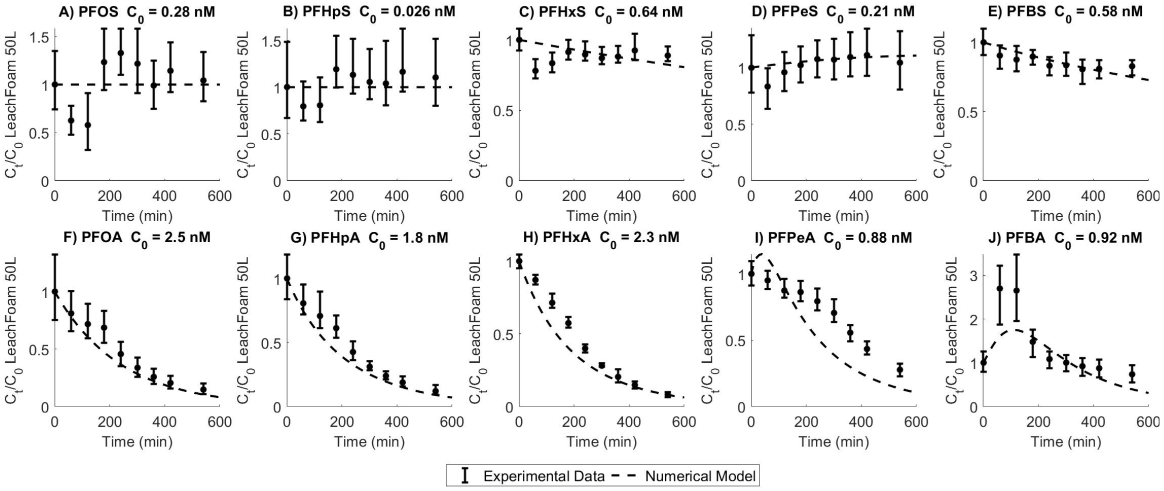

Figure S11. Individual degradation of PFSA and PFCA with chain lengths up to 8 for the EO run with 50 L leachate foam, using the kinetic constants calibrated based on the results from this experiment. Error bars represent min and max values based on the experimental and analytical duplicates (i.e. n = 4), dots represent the means and the dotted line is the model prediction with calibrated kinetic constants, see Table S7.

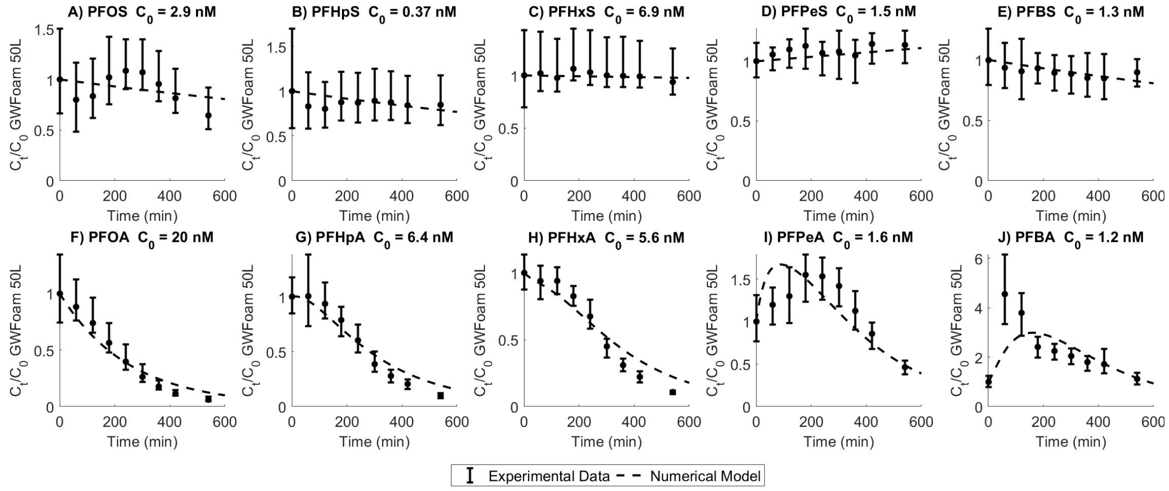

Figure S12. Individual degradation of PFSA and PFCA with chain lengths up to 8 for the EO run with 50 L groundwater foam, using the kinetic constants calibrated based on the results from this experiment. Error bars represent min and max values based on the experimental and analytical duplicates (i.e. n = 4), dots represent the means and the dotted line is the model prediction with calibrated kinetic constants, see Table S7.

REFERENCES

(1) Schaefer, C. E.; Andaya, C.; Burant, A.; Condee, C. W.; Urtiaga, A.; Strathmann, T. J.; Higgins, C. P. Electrochemical treatment of perfluorooctanoic acid and perfluorooctane sulfonate: Insights into mechanisms and application to groundwater treatment. *Chem. Eng. J.* **2017**, *317*, 424–432.

(2) Casas, G.; Martínez-Varela, A.; Roscales, J. L.; Vila-Costa, M.; Dachs, J.; Jiménez, B. Enrichment of perfluoroalkyl substances in the sea-surface microlayer and sea-spray aerosols in the Southern Ocean. *Environ. Pollut.* **2020**, *267*, 115512.

(3) Smith, S. J.; Wiberg, K.; McCleaf, P.; Ahrens, L. Pilot-scale continuous foam fractionation for the removal of per- and polyfluoroalkyl substances (PFAS) from landfill leachate. *ACS EST Water* **2022**, *2*, 841–851.

(4) Schultes, L.; Vestergren, R.; Volkova, K.; Westberg, E.; Jacobson, T.; Benskin, J. P. Per- and polyfluoroalkyl substances and fluorine mass balance in cosmetic products from the Swedish market: Implications for environmental emissions and human exposure. *Environmental Science: Processes and Impacts* **2018**, *20*, 1680–1690.

(5) Ateia, M.; Maroli, A.; Tharayil, N.; Karanfil, T. The overlooked short- and ultrashort-chain poly- and perfluorinated substances: A review. *Chemosphere* **2019**, *220*, 866–882.

(6) McDonough, C. A.; Higgins, C. P.; Choyke, S.; Barton, K. E.; Mass, S.; Starling, A. P.; Adgate, J. L. Unsaturated PFOS and other PFASs in human serum and drinking water from an AFFF-impacted community. *Environmental Science and Technology* **2021**, *55*, 8139–8148.

(7) McCord, J. P.; Strynar, M. J.; Washington, J. W.; Bergman, E. L.; Goodrow, S. M. Emerging chlorinated polyfluorinated polyether compounds impacting the waters of southwestern new jersey identified by use of nontargeted analysis. *Environmental Science and Technology Letters* **2020**, *7*, 903–908.

(8) Kärrman, A.; Yeung, L. W.; Spaan, K. M.; Lange, F. T.; Nguyen, M. A.; Plassmann, M.; De Wit, C. A.; Scheurer, M.; Awad, R.; Benskin, J. P. Can determination of extractable organofluorine (EOF) be standardized? First interlaboratory comparisons of EOF and fluorine mass balance in sludge and water matrices. *Environmental Science: Processes and Impacts* **2021**, *23*, 1458–1465.

(9) Hamers, T.; Kortenkamp, A.; Scholze, M.; Molenaar, D.; Cenijn, P. H.; Weiss, J. M. Transthyretin-binding activity of complex mixtures representing the composition of thyroid-hormone disrupting contaminants in house dust and human serum. *Environ. Health Perspect.* **2020**, *128*, 1–15.

(10) Verweij, W.; Durand, A.; Maas, J.; Van der Grinten, E. *Protocols belonging to the report 'Toxicity measurements in concentrated water samples '*; tech. rep.; 2010.

(11) Daubner, S. C.; Astorga, A. M.; Leisman, G. B.; Baldwin, T. O. Yellow light emission of vibrio fischeri strain Y-1 : purification and characterization of the energy-accepting yellow fluorescent protein. *Proc. Natl. Acad. Sci. U.S.A.* **1987**, *84*, 8912–8916.

(12) Radjenovic, J.; Duinslaeger, N.; Avval, S. S.; Chaplin, B. P. Facing the Challenge of Poly- and Perfluoroalkyl Substances in Water: Is Electrochemical Oxidation the Answer? *Environ. Sci. Technol.* **2020**, *54*, 14815–14829.
